# PARIS induced defects in mitochondrial biogenesis drive dopamine neuron loss under conditions of parkin or PINK1 deficiency

**DOI:** 10.1186/s13024-020-00363-x

**Published:** 2020-03-05

**Authors:** Sheila K. Pirooznia, Changqing Yuan, Mohammed Repon Khan, Senthilkumar S. Karuppagounder, Luan Wang, Yulan Xiong, Sung Ung Kang, Yunjong Lee, Valina L. Dawson, Ted M. Dawson

**Affiliations:** 1 Neuroregeneration and Stem Cell Programs, Institute for Cell Engineering, Johns Hopkins University School of Medicine, 733 North Broadway, Suite 731, Baltimore, MD 21205 USA; 2 Departments of Neurology, Iowa City, USA; 3 Adrienne Helis Malvin Medical Research Foundation, New Orleans, LA 70130-2685 USA; 4 Diana Helis Henry Medical Research Foundation, New Orleans, LA 70130-2685 USA; 5 Department of Anatomy and Physiology, College of Veterinary Medicine, Kansas State University, Manhattan, KS 66506 USA; 6 Departments of Physiology, Baltimore, USA; 7 Solomon H. Snyder Department of Neuroscience, Baltimore, USA; 8 Department of Pharmacology and Molecular Sciences, Johns Hopkins University School of Medicine, Baltimore, MD 21205 USA

**Keywords:** PARIS, ZNF746, Parkin, PINK1, Mitochondria, Parkinson’s disease, Dopaminergic neurons, Climbing defects

## Abstract

**Background:**

Mutations in PINK1 and parkin cause autosomal recessive Parkinson’s disease (PD). Evidence placing PINK1 and parkin in common pathways regulating multiple aspects of mitochondrial quality control is burgeoning. However, compelling evidence to causatively link specific PINK1/parkin dependent mitochondrial pathways to dopamine neuron degeneration in PD is lacking. Although PINK1 and parkin are known to regulate mitophagy, emerging data suggest that defects in mitophagy are unlikely to be of pathological relevance. Mitochondrial functions of PINK1 and parkin are also tied to their proteasomal regulation of specific substrates. In this study, we examined how PINK1/parkin mediated regulation of the pathogenic substrate PARIS impacts dopaminergic mitochondrial network homeostasis and neuronal survival in *Drosophila*.

**Methods:**

The UAS-Gal4 system was employed for cell-type specific expression of the various transgenes. Effects on dopamine neuronal survival and function were assessed by anti-TH immunostaining and negative geotaxis assays. Mitochondrial effects were probed by quantitative analysis of mito-GFP labeled dopaminergic mitochondria, assessment of mitochondrial abundance in dopamine neurons isolated by Fluorescence Activated Cell Sorting (FACS) and qRT-PCR analysis of dopaminergic factors that promote mitochondrial biogenesis. Statistical analyses employed two-tailed Student’s T-test, one-way or two-way ANOVA as required and data considered significant when *P* < 0.05.

**Results:**

We show that defects in mitochondrial biogenesis drive adult onset progressive loss of dopamine neurons and motor deficits in *Drosophila* models of PINK1 or parkin insufficiency. Such defects result from PARIS dependent repression of dopaminergic PGC-1α and its downstream transcription factors NRF1 and TFAM that cooperatively promote mitochondrial biogenesis. Dopaminergic accumulation of human or *Drosophila* PARIS recapitulates these neurodegenerative phenotypes that are effectively reversed by PINK1, parkin or PGC-1α overexpression in vivo. To our knowledge, PARIS is the only co-substrate of PINK1 and parkin to specifically accumulate in the DA neurons and cause neurodegeneration and locomotor defects stemming from disrupted dopamine signaling.

**Conclusions:**

Our findings identify a highly conserved role for PINK1 and parkin in regulating mitochondrial biogenesis and promoting mitochondrial health via the PARIS/ PGC-1α axis. The Drosophila models described here effectively recapitulate the cardinal PD phenotypes and thus will facilitate identification of novel regulators of mitochondrial biogenesis for physiologically relevant therapeutic interventions.

## Background

Parkinson’s disease (PD) is a chronic neurodegenerative movement disorder pathologically characterized by the deficiency of brain dopamine (DA) content and selective degeneration of substantia nigra DA neurons [1]. Although the majority of PD cases are considered sporadic, the few familial forms known to date contribute to at least 10% of the disease burden. Mutations in the genes encoding α*-synuclein*, *leucine rich repeat kinase 2* (*LRRK2*), *parkin*, *PINK1* and *DJ1* among others play a causative role in the development of PD with varying penetrance [2]. A general involvement of mitochondrial dysfunction in PD pathogenesis is reinforced by genetic and functional studies of pathogenic variants of familial PD genes [3]. Among the PD genes, the most compelling mitochondrial link exists for PTEN Induced Kinase 1 (PINK1) and parkin whose functions converge in common signaling pathways to regulate multiple domains of mitochondrial network homeostasis and quality control [4]. Besides the mitochondria, both PINK1 and parkin are localized to various other cellular compartments including the cytosol and exert neuroprotective functions [5–13].

Important clues to understanding the genetic link between parkin and PINK1 as well as their role in maintaining mitochondrial integrity stem from studies in *Drosophila*. While parkin or PINK1 deficient *Drosophila* exhibit marked muscle and germ-line pathologies resulting from dysfunctional fission/fusion dynamics [14–20], reports on DA neuron degeneration in these mutants is variable depending on the approach (reviewed in [4]). Substantial work in the last 5 to 10 years has focused on the role of parkin and PINK1 in mediating mitophagy [3, 21, 22]. Despite evidence that parkin and PINK1 coordinate mitophagy in cell culture systems overexpressing the said proteins, there is very little evidence that the loss of DA neurons is due to defects in mitophagy [23]. For instance, defects in basal mitophagy were not observed in parkin or PINK1 knockout (KO) *Drosophila* expressing mitophagy reporters in a variety of cell types including DA neurons despite the loss of DA neurons observed by some studies in these models [24–26]. PINK1 KO or parkin S65A knock-in mice expressing a mitophagy reporter also failed to show defects in basal mitophagy [27, 28]. Combining mito-Keima imaging with correlative light and electron microscopy (CLEM) in *Drosophila* indicates that mitophagy may occur in vivo and that the absence of parkin or PINK1 reduced basal mitophagy [14, 24, 29, 30]. However, 1 week old PINK1 or parkin KO *Drosophila*, which already exhibit mitochondrial abnormalities [14, 29] fail to exhibit a significant reduction in mitophagy [30] suggesting that the mitochondrial deficits are separate from the mitophagy defects. What then might account for the mitochondrial deficits and loss of DA neurons?

Several co-substrates of PINK1 and parkin are now known and are thought to regulate different aspects of mitochondrial quality control [4, 22, 31]. PARIS (ZNF746) is one such substrate whose cellular levels are regulated by PINK1 mediated phosphorylation and parkin dependent ubiquitination [13, 32]. A mitochondrial link for PARIS emanates from the transcriptional repressive effect it has on peroxisome proliferator-activated receptor gamma co-activator 1-alpha (PGC-1α), a co-activator that serves as a nodal regulator of mitochondrial biogenesis and antioxidant response [32, 33]. Germline ablations of PINK1 or parkin in mouse or *Drosophila* models leave mitochondrial homeostasis in DA neurons largely unperturbed due to developmental or epistatic compensation [4, 22]. Conditional inactivation of parkin or PINK1 in mouse ventral midbrain, however, leads to DA neuronal loss that is driven by PARIS accumulation [13, 32, 34].

To explore if PARIS dependent mitochondrial perturbations could trigger DA neuron loss under conditions of reduced PINK1 or parkin activity in *Drosophila* models, we generated transgenic *Drosophila* lines expressing human PARIS (hereafter referred to as PARIS) and a transcriptionally inactive human PARIS mutant (C571A). We find that ubiquitous expression of PARIS but not the C571A mutant is selectively toxic to dopaminergic neurons in *Drosophila* and leads to progressive DA neuron loss with concomitant climbing deficits. Such defects are reminiscent of those resulting from shRNA mediated reductions in dopaminergic PINK1 or parkin activity. By employing Fluorescence Activated Cell Sorting (FACS), we further demonstrate that PARIS accumulation within the DA neurons leads to transcriptional repression of key mediators of mitochondrial biogenesis that is reversed by overexpression of PINK1, parkin or PGC-1α. Accumulations of the single *Drosophila* homolog of PARIS (dPARIS) within the DA neurons also exerts similar dopaminergic neurotoxicity by impinging on mitochondrial biogenesis, highlighting the evolutionarily conserved nature of the PINK1/parkin/ PGC-1α axis in promoting DA neuron survival. Together, our studies uncover an essential requirement for mitochondrial biogenesis in maintaining mitochondrial network homeostasis within the DA neurons. The *Drosophila* models of PARIS we describe here will further facilitate screening for novel regulators of mitochondrial biogenesis that could each serve as nodal points for therapeutic access in PD therapy to preserve mitochondrial homeostasis in dopamine neurons.

## Methods

### Cell lines

*Drosophila* S2 were routinely maintained at a density of 2–3 × 10^6^ cells/ml in M3 + BYPE media (pH 6.6) supplemented with Fetal Bovine Serum (HyClone™) and Penicillin-Streptomycin solution (Sigma). The M3 + BYPE media contained 39.4 g/L of Shields and Sang powdered medium (Sigma), 0.5 g/L of Potassium bicarbonate (Sigma), 1 g/L yeast extract (Sigma), 2.5 g/L bactopeptone (Difco). Cell cultures and transfected cells were maintained at 25 °C without CO_2_.

### Fly stocks and maintenance

*Drosophila melanogaster* fly stocks were handled using standard protocols, maintained in a 12 h light/dark cycle and fed *Drosophila* standard diet consisting of cornmeal, agar, yeast, sucrose, and dextrose. All experimental crosses were kept at 25 °C. In all experiments, both male and female flies were used. Transgenic stocks and Gal4 lines were obtained from the Bloomington *Drosophila* Stock Center (BDSC), the Vienna *Drosophila* RNAi stock center (VDRC), the FlyORF or were gifts (see Additional file 1, Table S1 for details). Human PARIS and C571A constructs were generated using standard cloning procedures by subcloning the respective cDNA sequence into pUAST plasmid (DGRC). Following sequence verification, constructs were microinjected into w^1118^ embryos (The BestGene, Inc). In each experiment, the relevant Gal4 heterozygous flies were used as control. Flies expressing shRNA targeting EGFP were used as control for all TRIP shRNA lines.

### Cloning and transfection procedures

*Drosophila* PARIS coding sequence was cloned using standard cloning procedures into the pAC5.1 V5-His-B vector (Life Technologies) in frame with the C-terminal V5/His tag in the vector using KpnI and EcoRV restriction sites. A FLAG tag was introduced at the N-terminus of *Drosophila* parkin (dparkin) coding sequence by PCR. The FLAG tagged and untagged dparkin coding sequence were independently cloned using standard procedures into pAC5.1 V5-His-B vector at XhoI and SacII sites. The primers used are listed in Additional file, Table S2. Transfections of S2 cells were carried out using X-tremeGENE™ HP DNA Transfection Reagent (Sigma) following manufacturer’s protocol. Transfected cells were maintained at 25 °C without CO_2_ for 72 h prior to harvesting cells for western blot analysis.

### Immunostaining

*Drosophila* brain dissection and immunostaining were performed as described previously with modifications [35]. Briefly, adult *Drosophila* brains were fixed in 4% paraformaldehyde for 30 min at room temperature (RT), washed three times in 1 X PBS containing 0.1% Triton X-100 (wash buffer), blocked in PBS containing 5% normal goat serum and 0.2% Triton X-100 for 40 min at RT. Brains were then incubated in primary antibody in blocking solution for at least 48 h at 4^o^ C, followed by three 10 min washes at RT. Secondary antibody was applied in blocking solution and samples incubated overnight at 4 °C. Following five 10 min washes at RT, brains were mounted on glass slides using ProLong Gold antifade mountant (Life Technologies). The primary antibodies used are as follows: 1:1000 anti-Tyrosine Hydroxylase antibody (Immunostar, cat no. 22941), 1:500 anti-serotonin (α-5HT; Sigma, cat no. S5545), 1:500 anti-GFP antibody (Millipore, cat no. AB10145). Secondary antibodies were goat anti-mouse, anti-rabbit or anti-Chicken Alexa fluorochromes at 1:1000 (Thermo Fisher, cat no. A-11032, A32731, A-11039).

### Imaging and quantification procedures

Confocal microscopy and image acquisition were performed with a Zeiss LSM710 laser scanning confocal microscope at 40X magnification. Images were scanned at 1024 X 1024 pixels, with a slice thickness of 1 μm and line and frame average of 4. Different channels were scanned sequentially to avoid bleed-through. Imaging settings were first determined for the control conditions in each experiment and these setting were maintained for the various genotypes. For DA or 5-HT neuron counts, stacked Z-projections of images were created and cell bodies stained with anti-TH (for DA neurons), anti-5HT (for serotonin neurons) or anti-GFP were manually counted. The investigator performing the neuronal counts was blinded to the individual genotype information. Integrated intensity of mito-GFP relative to TH was measured using the ImageJ software (NIH). TH intensity in the 568 nm channel (marking DA neurons) was used to select regions of interest (ROI). These ROIs were then transferred to the 488 nm channel to measure fluorescence intensity of mito-GFP labelled mitochondria within the same ROIs.

### L-DOPA treatment

Flies were maintained in batches of 20 on standard fly media containing 1 mg/mL of L-DOPA (Sigma) in DH2O. A small aliquot (~ 200 ul) of L-DOPA was added to the surface of fly media and allowed to absorb for at least 6 h prior to transferring flies. Flies were subjected to L-DOPA treatment for 3 days prior to climbing assays and transferred to fresh fly media containing L-DOPA every 3 days for the longevity assays.

### Climbing and longevity assays

Climbing assay were performed as described previously [36]. Flies were collected immediately following eclosion and maintained in batches of 20 at 25^o^ C in vials containing standard food media. Each batch of flies was transferred to an empty 9 cm fly vial (Genesee Scientific, cat no. 32–113) with a line drawn 6 cm from the bottom. Flies were tapped to the bottom to induce an innate climbing response. The number of flies that crossed the 6 cm mark in 15 s were counted. Five to ten technical replicates were performed for each batch to ensure an accurate reading at each time-point and average of the independent trials was used to calculate the percentage of flies with climbing defects. Flies were assayed every 10 days from eclosion until day 50. For longevity assay, F1 progeny from the respective Actin-Gal4 crosses were collected immediately following eclosion and maintained at 25 °C on standard food media. 100 male and female flies were maintained separately in batches of 20 flies per vial and transferred to fresh food vials every 3 days. Kaplan Meier curves and statistical analysis was performed according to GraphPad Prism 8 survival function, with a Log-rank Mantel Cox test to determine statistical significance.

### Calculation of eclosion rate

Eclosion rate was calculated as described previously [37]. Female virgin UAS-PARIS flies were crossed with male Actin-Gal4/Cyo flies to generate heterozygous Act>PARIS expressor flies or PARIS/Cyo controls. The F1 progeny were scored 10 days after the parent crosses were set. The proportion of flies eclosing was calculated as the number of Act>PARIS flies eclosed divided by the total number of flies eclosed including the Cyo internal control. For the control genotypes, this was normalized to 100%. For additional genotypes tested, e.g. PARIS in combination with parkin, the experimental condition (Act>PARIS, parkin) was compared against the control condition without PARIS, e.g. Act>parkin.

### Immunoprecipitation and Immunoblot analysis

Heads collected from cold anaesthetized flies were homogenized in lysis buffer (20 mM HEPES, 100 mM KCl, 5% glycerol, 10 mM EDTA, 0.1% Triton, 1 mM DTT) supplemented with EDTA-free protease inhibitor cocktail (Roche) using a micro tissue grinder. Following a 30 min incubation on ice, samples were centrifuged at 12000 g for 10 min. The supernatant was mixed in a 1:1 ratio with 2X Laemmli sample buffer (Biorad) containing 2-mercaptoethanol. S2 cells were lysed by mixing with S2 lysis buffer (50 mM Tris pH 7.5; 150 mM NaCl; 5 mM EDTA; 1% NP-40) supplemented with EDTA-free protease inhibitor cocktail (Roche). Following 15 min incubation on ice, samples were centrifuged at 12000 g for 10 min and supernatant mixed in a 1:1 ratio with 2X Laemmli sample buffer (Biorad, 1,610,737) containing 2-mercaptoethanol. Protein concentrations were estimated using BCA protein assay kit (Thermo Fisher). Equivalent amounts of S2 lysates were used in co-immunoprecipitation experiments. Anti-FLAG M2 magnetic beads (Sigma), anti-V5-tag mAb magnetic beads (MBL International) or anti-dPARIS conjugated Dynabeads protein G (Thermo Fisher) were used as per manufacturer’s protocol for FLAG, V5 or dPARIS pull down experiments, respectively. Following an overnight incubation of respective lysates with 40 μl of FLAG/V5 beads or 20 μl of anti-dPARIS conjugated Dynabeads, the beads were washed thrice with S2 lysis buffer and bound proteins were eluted in 1X Laemmli sample buffer containing 2-mercaptoethanol.

Equivalent amounts of protein were electrophoresed in 10% tris-glycine SDS-PAGE gel, transferred to Hybond ECL nitrocellulose membrane (GE Healthcare). Membranes were blocked for 1 h using phosphate buffered saline (PBS) supplemented with 0.05% Triton X-100 and 5% non-fat milk followed by overnight incubation at 4 °C in PBS supplemented with 0.05% Triton X-100 and corresponding primary antibody. Human PARIS, PARIS transcriptional mutant (C571A) and PARIS phosphomutant (PARIS DM) levels were detected using anti-PARIS antibody (1:2000, NIH NeuroMab, cat no. 75–195). *Drosophila* PARIS (dPARIS) was detected using a polyclonal antibody we generated using services from Thermo Fisher and that we subsequently characterized for specificity in immunoblot and fluorescent applications. Anti-FLAG M2 (Sigma, cat no. F3165) or V5-tag (Cell Signaling, cat no. 13202S) antibodies were used for detection of FLAG or V5 tagged proteins respectively. Phosphorylation of dPARIS was detected using anti-phosphoserine antibody (Abcam, cat no. ab9332). Anti-Ubiquitin antibody (1B4-UB, Abcam, cat no. ab122) was used to detect polyubiquitin modifications of dPARIS. Actin used as loading control was detected using anti-β-actin antibody (1:10000, Abcam, cat no. ab49900). Anti-Mouse IgG or anti-Rabbit IgG horseradish peroxidase-conjugated secondary antibody (1:2000, Cell Signaling, cat no. 7076 and 7074) was used to detect protein bands by electrochemiluminescence. Images were collected digitally and band intensities were quantified by densitometry using ImageJ software (NIH) and normalized to the loading control.

### Measurement of DA and serotonin level by HPLC

Biogenic amines in the fly samples were measured by High-performance liquid chromatography with electrochemical detection (HPLC-ECD) [38]. Briefly, fly heads (50 heads/group) were quickly removed, minced and sonicated in ice-cold 0.01 mM perchloric acid (containing 0.01% EDTA and 60 ng 3, 4-dihydroxybenzylamine (DHBA) as an internal standard). The samples were centrifuged at 15,000×*g*, 30 min, 4 °C, then the supernatant was passed through a 0.2 μm filter. The samples injected by rheodyne were split into two different columns to measure monoamine and its metabolite. The HPLC instrument equipped with two 3 μm, C18 column as follow, ALF 105 (50 × 1.0 mm, Antec Leyden, Netherland) for analysis of NE, DA and 5HT and ALF-115 column, (150 × 1.0 mm, Antec leyden, Netherland) for analysis of metabolites. The separated samples were detected by dual electrochemical detector (Model Decade II, Antec Leyden, Netherland). The protein concentrations of tissue homogenates were measured using the BCA protein assay kit (Thermo Scientific). Data were normalized to protein concentrations (ng neurotransmitters/ mg tissue).

### Fluorescence activated cell sorting (FACS) of DA neurons

Transgenic fly lines carrying the TH-Gal4; UAS-GFP constructs were crossed with flies carrying the respective UAS construct. F1 progeny that express GFP and the respective transgene in the DA neurons in each case were collected and aged for 20 days to be used for FACS analysis as previously described with modifications [39]. Fifty adult brains were dissected and mechanically dissociated in TrypLE Express (Gibco) by incubating at 37^o^ C for 40 min followed by passage through a syringe fitted with a 29-gauge needle 5–10 times. Flies were maintained at room temperature during brain dissections in order to avoid temperature induced induction of gene expression in the DA neurons. Following dissociation, cells were pelleted at low speed, resuspended in DMEM media (Gibco) supplemented with 10% fetal bovine serum (Hyclone) and passed through a 70 μM mesh strainer. In order to label live cells, the membrane permeant Vybrant DyeCycle Ruby stain (Life Technologies) was applied to the cell suspension prior to sorting. Cells were maintained at 4^o^ C until flow analysis and sorting that was always performed within 30 min of completion of the protocol.

Cells were subjected to FACS using a SH800 (Sony, IL) flow cytometer fitted with a 100 μM nozzle at 20 psi. GFP and DyeCycle Ruby detection was done using a 488 nm and 635 nm laser, respectively. Samples were sorted at 4^o^ C and the sorted cells were collected in 0.5 ml of Trizol-LS (Invitrogen) or DMEM media supplemented with 10% Fetal bovine serum (Hyclone) for RNA and DNA isolation, respectively. The yield of GFP-positive cells ranged from 2000 to 2500 cells/sort per 50 adult fly brains.

### RNA and DNA extraction from FACS neurons

RNA extraction from DA neurons post cell sorting was performed using the Trizol LS Reagent (Invitrogen) according to the manufacturer’s instructions. Dnase-I (Invitrogen) treated RNA was used for cDNA preparation using the Superscript III first-strand synthesis system (Thermo Fisher) following manufacturer’s protocol.

Genomic DNA extraction from DA neurons post cell sorting was performed by pelleting FACS sorted neurons by centrifugation at 4000 rpm for 10 min. The pellet was resuspended in 100 μl of 1 X PBS containing 20 mM EDTA followed by addition of 100 μL of 2 X Proteinase K buffer (100 mM Tris-HCL, pH 7.5, 50 mM EDTA, 2% SDS). The sample was vortexed and proteinase K was added to final concentration of 500 μg/ml and incubated overnight at 56 °C. An equal volume of phenol/chloroform/isoamyl alcohol was added to the DNA solution. DNA was precipitated by adding 1/10 volume of 3 M sodium acetate, pH 5.2 and 1 volume 100% EtOH followed by centrifugation at 13000 g for 10 min. The DNA pellet was washed with 70% ethanol, air-dried and re-suspended in 20 μl of distilled water,

### Real time quantitative PCR

Real-time quantitative PCR was performed on an Applied Biosystems ViiA 7 Real-Time PCR System using the Maxima SYBR Green/ROX qPCR Master Mix (Thermo Fisher). Real-time PCR reactions were carried out in duplicates in 20 μl reaction volumes containing 2 μl cDNA template and 1.5 μM each of forward and reverse primer. Reactions were performed in 384 well plates for 40 amplification cycles at 95 °C for 15 s, 60 °C for 45 s, and 72 °C for 1 min with plate readings recorded after each cycle. Threshold cycle (Ct) values were obtained, and the ΔΔCT method was used to calculate the fold change in transcript level of the sample relative to the control. RP49 was used as an internal standard and reference gene. Primers used are listed in Additional file 2, Table S2**.**

### Measurement of mtDNA density

The relative number of mtDNA genomes per diploid nuclear genome for the different genotypes was determined by quantitative real-time PCR as described previously [40]. Genomic DNA was isolated from FACS sorted DA neurons and mtDNA was quantified by amplifying a 105 bp region of the mitochondrial large ribosomal RNA gene (lrRNA, CR34094) or a 249 bp region of the mitochondrial cytochrome B gene (CytB, CG34090). Nuclear genomic DNA was quantified by amplifying a 215 bp region of the single-copy nuclear gene that encodes the 215 kDa subunit of RNA polymerase II (rpII215; CG1554). The amount of mitochondrial DNA (mtDNA) was assessed by the ratio of mtDNA to nuclear DNA (nuDNA) copy number. Primers used are listed in Additional file 2, Table S2**.**

### Quantification and statistical analysis

Statistical analyses were performed using GraphPad statistical software (Graphpad Prism, version 8, San Diego, CA). The criteria for significance was: ns (not significant) *p* > 0.05, ∗*p* < 0.05, ∗∗*p* < 0.01, ∗∗∗*p* < 0.001, ****p < 0.0001. Significant differences between 2 groups were analyzed using a two-tailed Student’s T-test. For more than 2 groups, a one-way ANOVA (with Tukey’s post hoc correction for multiple comparisons) was used. For mixed groups, a two-way ANOVA (with Tukey’s post hoc correction for multiple comparisons) was used. The graph representation, definition of n and which statistical test was performed is indicated in the figure legend. Error bars show standard error of the mean (SEM) or standard deviation (SD), as indicated in the figure legend. Sample size was chosen according to that used for similar experiments in the literature.

## Results

### Characterization of wild type and mutant PARIS transgenic flies

**Fig. 1:**
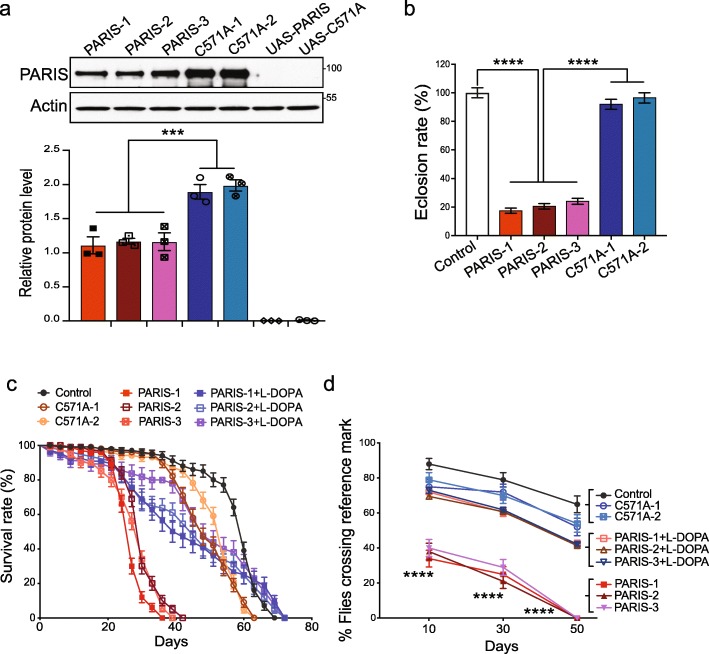
Ubiquitous expression of PARIS in *Drosophila* results in partial lethality, shortened lifespan and climbing defects. **a** Immunoblot analysis of PARIS and PARIS mutant (C571A) protein levels in independent transgenic fly lines as indicated, *N* = 3. **b** Actin-Gal4 driven ubiquitous expression of PARIS causes partial lethality during development while C571A has no lethal effects. Eclosion rate normalized to control (Act-Gal4/+). **c** Kaplan Meier survival curve showing substantially reduced life span in Act>PARIS flies compared to Act-Gal4/+ control. Also see Additional file 3, Table S3 (**d**) Ubiquitous expression of PARIS but not C571A leads to a significant and age-related progressive decline in climbing performance that is restored by L-DOPA treatment. Act-Gal4/+ flies served as control. *N* = 60–80 flies per group. Quantitative data = mean ± SEM, One-way ANOVA ***p < 0.001, ****p < 0.0001


Multiple independent transgenic UAS lines were generated through P-element mediated germ-line transformation. The bipartite UAS-Gal4 system [41] was used to target expression of the PARIS protein and a transcriptionally inactive PARIS mutant (C571A) to different fly tissues and cell types. In order to examine the physiological effects of PARIS and the PARIS mutant in vivo, the UAS- PARIS and UAS-C571A lines were crossed to actin-Gal4 driver, which exhibits strong, ubiquitous Gal4 activity throughout development [42]. Three independent lines of PARIS and two C571A lines with equivalent expression of PARIS or the C571A mutant, respectively were identified using immunoblot analysis (Fig. 1a) and advanced for further analysis. Immunoblot analysis indicates that while wild type PARIS is expressed at high levels under the direction of the actin-Gal4 driver, C571A is expressed at almost two-fold higher levels than wild type PARIS (Fig. 1a). Expression of wild type PARIS with the actin-GAL4 driver causes approximately 80% lethality during development, whereas C571A has no effect (Fig. 1b). Wild type PARIS flies that eclosed exhibit shorter longevity with median survival of 29 days compared to median survival of 51 days and 60 days observed for C571A and control flies (Act-Gal4/+), respectively (Fig. 1c and Additional file 3, Table S3). Act>PARIS flies also exhibit an age-related decline in climbing performance with complete loss of climbing ability at 50 days. In contrast, Act>C571A flies exhibit a decline in climbing performance that is similar to the Act-Gal4/+ control flies (Fig. 1d, Additional file 4, Video-1). In order to examine if the motor deficits in the Act>PARIS flies result from DA deficiency, the Act>PARIS flies were subjected to L-DOPA treatment. Administration of L_DOPA significantly improved climbing performance in these flies in the different age groups assayed, indicating that the progressive motor impairments result from DA depletion (Fig. 1d, Additional file 5, Video-2). L-DOPA treatment also improved longevity in the PARIS flies with a median survival of 45 days (Fig. 1c).

**Additional file 5: Video-2.** Effect of L-DOPA treatment on climbing performance in PARIS flies. Compared to untreated Act>PARIS flies, L-DOPA treatment significantly restores climbing performance in Act>PARIS flies to control (Act/+) levels (MP4).


To examine if the developmental lethality caused by PARIS accumulation is regulated by parkin/PINK1 signaling, genetic rescue experiments were conducted by expressing the human or *Drosophila* homolog of parkin or PINK1 in the PARIS background. Actin-Gal4 driven expression of the human (hparkin) or fly parkin (dparkin) partially rescues the lethal effect in the PARIS flies. However, Actin-Gal4 driven overexpression of human or fly PINK1 by itself caused complete lethality which abrogated further evaluation of the relative effect of PINK1 in the PARIS flies. Ubiquitous expression of PARIS in parkin or PINK1 null background also caused complete lethality (Additional file 6, Table S4). Despite several attempts, we were unable to generate homozygous fly lines that would allow for dopaminergic expression of PARIS in the parkin (Additional file 7, Table S5) or PINK1 null background which prevented use of the parkin or PINK1 null mutants to further test genetic epistasis.

### Expression of PARIS leads to progressive age-dependent loss of DA neurons

**Fig. 2:**
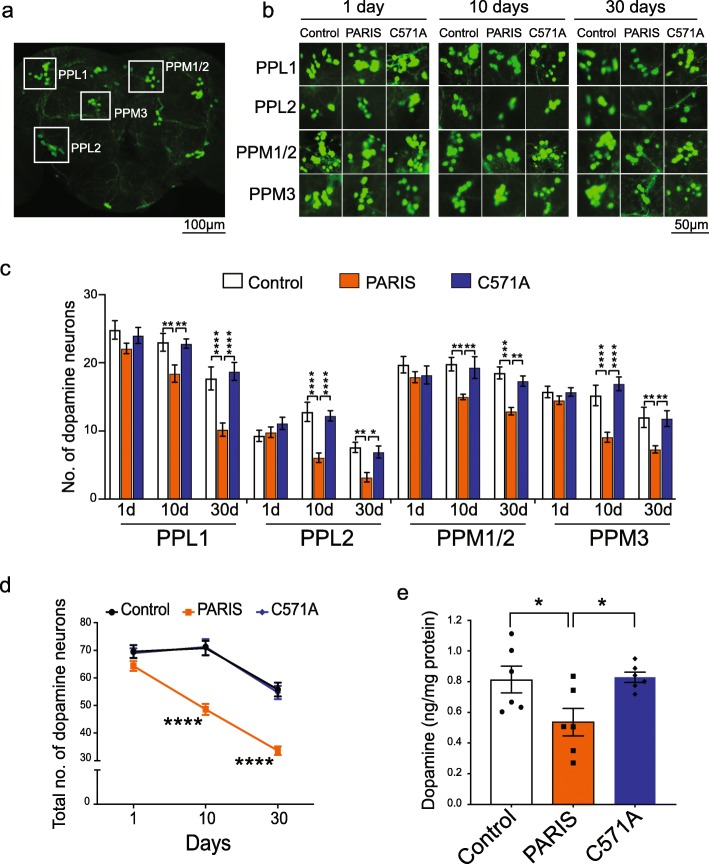
Ubiquitous expression of PARIS causes progressive DA neuron degeneration. **a** Representative confocal image of the adult *Drosophila* brain stained with anti-TH showing the location of the major DA neuron clusters PPL1, PPL2, PPM1/2, and PPM3. Scale = 100 μM. **b** Representative confocal images showing individual DA neuron clusters in control (Act-Gal4/+), PARIS and C571A flies at the indicated time points. Scale = 50 μM. **c** Quantification of DA neuron number in the indicated dopaminergic cluster in flies ubiquitously expressing PARIS on days 1, 10 and 30 shows progressive loss of DA neurons compared to C571A and control flies (Act-Gal4/+), *N* = 10 flies per age group for the indicated genotypes. **d** Total number of DA neurons in the PPL1, PPL2, PPM1/2, and PPM3 clusters reveal age-related progression of neuron loss in PARIS but not C571A flies. **e** High performance liquid chromatography (HPLC) analysis of DA levels in fly heads shows significant reduction in DA content in PARIS but not in C571A flies compared to Act-Gal4/+ controls. Quantitative data = mean ± SEM, One-way ANOVA *p < 0.05, **p < 0.01, ****p < 0.0001. See also Additional file 8, Figure S1

We next examined if ubiquitous expression of PARIS can lead to age-related neurodegeneration in vivo. Actin-Gal4 directed expression of PARIS leads to progressive and selective loss of DA neurons in the major DA neuronal clusters PPL1, PPL2, PPM1/2, and PPM3 (Fig. 2a-d). A significant reduction in the number of neurons within all the dopaminergic clusters is observed in 10-day old PARIS (Act>PARIS) flies with a trend towards further reduction at 30 days (Fig. 2b-d). However, no difference in DA neuron number within individual clusters is observed between the control (Act-Gal4/+), Act>PARIS and Act>C571A flies on day 1 immediately after eclosion (Fig. 2b-d). Thus, PARIS expression causes adult-onset degeneration of DA neurons that progresses with age. Accompanying the loss of DA neurons in the PARIS flies is a reduction in DA content as assessed by high performance liquid chromatography (HPLC) (Fig. 2e). DA neuron number in Act>C571A is comparable to the control flies (Act-Gal4/+) at all time points (Fig. 2b-d). There is also no difference in DA content between Act>C571A and control flies (Act-Gal4/+) (Fig. 2e). Immunostaining of serotonergic neurons with an anti-5-hydroxytryptamine (5-HT) antibody does not reveal any reduction in serotonin neurons between control (Act-Gal4/+) and Act>PARIS transgenic flies (Additional file 8, Figure S1a-b). HPLC analysis additionally shows equivalent levels of serotonin in these flies (Additional file 8, Figure S1c). The overall morphology of the brain was unaffected by expression of PARIS (data not shown) indicating that PARIS does not cause wide spread neurodegeneration, rather the PARIS induced degenerative effects are specific to DA neurons.

To determine if the expression of PARIS is toxic to other tissues, the structure of wings, muscle, and eye were examined and appeared unaffected (data not shown). To exclude the possibility that the climbing defect in the PARIS flies was due to a muscle defect, PARIS was selectively expressed in muscle using the Mef2-Gal4 driver [43]. Climbing performance in the Mef2 > PARIS flies is however, comparable to the control flies (Mef2-Gal4/+) (Additional file 8, Figure S1d). Furthermore, electron microscopy analysis reveals no substantial abnormalities in the ultrastructure of muscle in the Mef2 > PARIS flies (Additional file 8, Figure S1e).

### Parkin, PINK1 and PGC-1α rescue dopaminergic neuron loss caused by PARIS accumulation

**Fig. 3:**
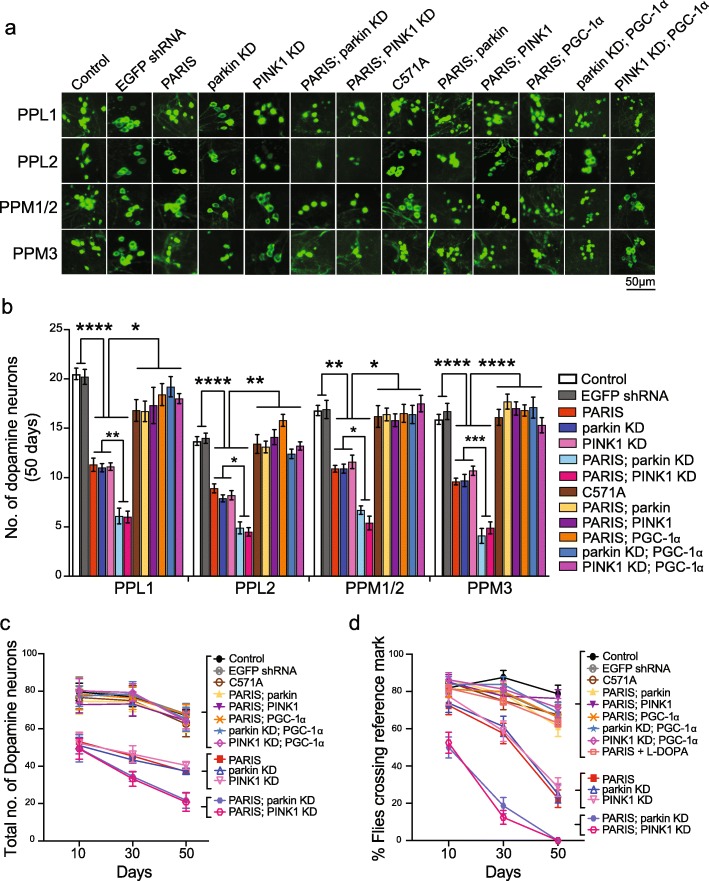
Rescue of PARIS induced dopaminergic neurodegeneration and climbing defect by PINK1 or parkin. **a** Representative confocal images of DA neuron clusters visualized by GFP immunofluorescence in 50-day old flies in the genotypes indicated. Scale = 50 μM. **b** TH-Gal4 driven PARIS, but not C571A causes significant loss of neurons in all DA neuron clusters on day 50, compared to age-matched control flies (TH > GFP). Knock-down of parkin or PINK1 in the PARIS flies phenocopies this effect and also exacerbates DA neuron loss in PARIS flies. PARIS induced neurodegenerative effects are ameliorated by overexpression of PINK1, parkin or PGC-1α. Overexpression of PGC-1α also rescues neuron loss in PINK1 or parkin knockdown flies. Quantitative data = mean ± SEM, One-way ANOVA *p < 0.05, **p < 0.01, ***p < 0.001, ****p < 0.0001. **c** Total number of DA neurons in the four major DA for the indicated time points. *N* = 10 flies per genotype for each time point. Quantitative data = mean ± SD, Two-way ANOVA. See also Tables S4 and S5 (**d**) Comparison of climbing performance in the different genotypes at the indicated time points. TH > GFP flies served as control. *N* = 80 flies per genotype for time points indicated. Quantitative data = mean ± SEM, Two-way ANOVA. See also Tables S6 and S7. TH-Gal4 mediated EGFP shRNA induction served as non-target control for shRNA response. See also Additional file 9, Figure S2 and Additional file 13, Figure S3


We next investigated the effect of PARIS expression specifically in DA neurons. Similar to the effects observed with the actin-Gal4 driver, expression of PARIS selectively in DA neurons using the DA neuron specific tyrosine hydroxylase Gal4 driver (TH-Gal4) [44] leads to an age-related progressive loss in the PPL1, PPL2, PPM1/2, and PPM3 DA neuron clusters (Fig. 3a-c; Additional file 9, Figure S2a-d; Additional files 10-11, Tables S6-S7). Dopaminergic expression of PARIS also leads to progressive decline in climbing performance with age that is effectively restored by L-DOPA treatment (Fig. 3d; Additional files 10-11, Table S6-S7; Additional file 12, Video-3). Selective expression of PARIS in the serotonergic or cholinergic neurons using the Trh-Gal4 [45] and Cha-Gal4 [46] drivers, respectively has no effect on the viability of these neurons or climbing performance (Additional file 13, Figure S3a-e). In addition, selective expression of PARIS in motor neurons with the D42-Gal4 driver [47] also has no effect on climbing behavior (Additional file 13, Figure S3f). Taken together, these data confirm that PARIS is selectively toxic to DA neurons in *Drosophila*.

**Additional file 12: Video-3.** Effect of L-DOPA treatment on climbing performance in TH > PARIS flies. Compared to untreated TH > PARIS flies, L-DOPA treatment restores climbing performance in TH > PARIS flies that is comparable to control flies (TH > GFP).


**Fig. 4:**
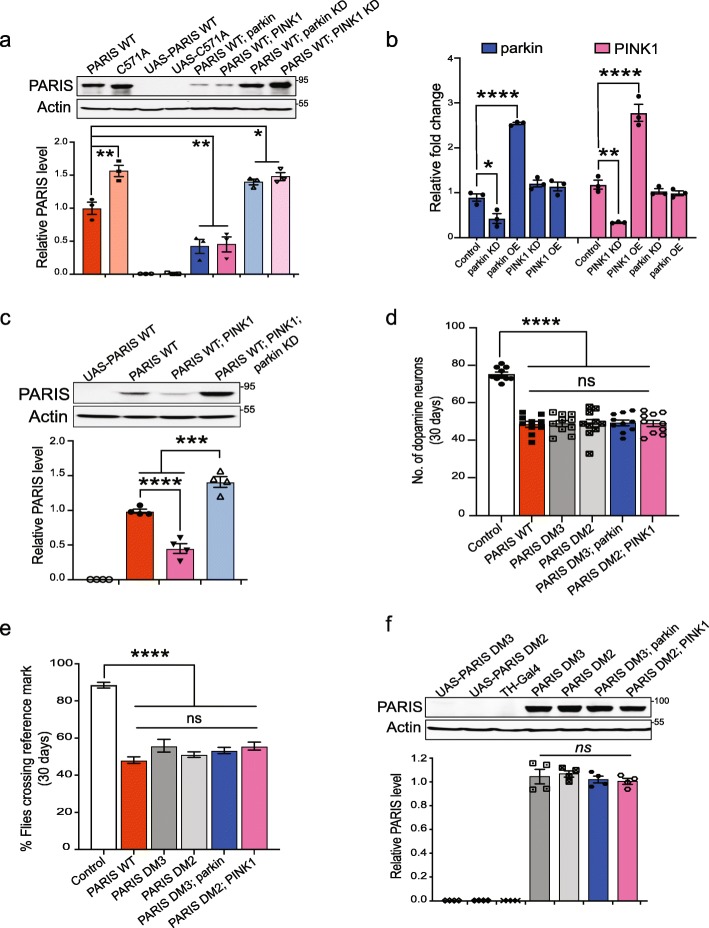
Regulation of dopaminergic levels of PARIS by PINK1 and parkin. **a** Immunoblot analysis and quantification of PARIS in flies expressing the indicated transgenes under the control of TH-Gal4 driver, *N* = 3. **b** Quantitative RT-PCR analysis in FACS sorted DA neurons showing PINK1 or parkin transcript levels upon TH-Gal4 mediated knockdown or overexpression of the respective genes. **c** Parkin functions downstream of PINK1 in a linear pathway to regulate PARIS levels in the DA neurons. Indicated transgenes expressed using TH-Gal4 driver, *N* = 4. **d** DA neuron loss in 30-day old flies expressing phosphodeficient PARIS double mutant (PARIS DM) is not rescued by dopaminergic overexpression of parkin or PINK1. *N* = 10 flies per indicated genotype. **e** Climbing defects in 30-day old TH > PARIS DM flies persist even under conditions of parkin or PINK1 overexpression in DA neurons. *N* = 60 flies per indicated genotype. **f** Dopaminergic overexpression of parkin or PINK1 does not attenuate accumulation of PARIS DM in DA neurons.TH-Gal4/+ flies served as control, *N* = 4. Quantitative data = mean ± SEM. One-way ANOVA, ns (non-significant), *p* > 0.05, *p < 0.05, **p < 0.01, ****p < 0.0001. Quantification of mRNA transcript levels relative to TH > GFP control from three independent FACS experiments each employing 50 fly brains of the indicated genotype. Data = mean ± SEM. Unpaired two-tailed Student’s t test *p < 0.05. See also Additional file 14, Figure S4

To determine whether loss of DA neurons following PARIS expression is regulated by parkin or PINK1, the effect of genetically manipulating parkin or PINK1 levels in the PARIS transgenic flies was examined. Dopaminergic overexpression of parkin or PINK1 prevents the loss of DA neurons and rescues climbing defects in the PARIS transgenic flies (Fig. 3a-d, Additional file 9, Figure S2a-d; and Additional files 10-11, Tables S6-S7). Targeted knockdown of either parkin or PINK1 using specific shRNA lines [48–50] in the DA neurons leads to loss of DA neurons and climbing defects and further exacerbates these dopaminergic phenotypes in the PARIS overexpressing flies (Fig. 3a-d Additional file 9, Figure S2a-d; and Additional files 10-11, Tables S6-S7). Immunoblot analysis reveals that dopaminergic knockdown of parkin or PINK1 leads to marked accumulation of PARIS whereas PARIS levels are significantly decreased in flies overexpressing parkin or PINK1 (Fig. 4a-b). Dopaminergic knock down or overexpression of parkin does not impact PINK1 transcript levels and vice versa (Fig. 4b), further supporting the specific nature of PARIS regulation by PINK1 and parkin. While PINK1 overexpression in the dopaminergic neurons abrogates PARIS accumulation, concomitant knock down of parkin prevents this effect (Fig. 4c) indicating that parkin functions downstream of PINK1 in a common pathway to regulate neuronal levels of PARIS. Indeed, parkin mediated ubiquitination and clearance of PARIS is primed by its phosphorylation by PINK1. In fact, dopaminergic expression of a phosphorylation deficient PARIS double mutant (PARIS DM) carrying S322A and S613A mutations [13] phenocopies the toxicity associated with wild type PARIS accumulation and leads to loss of DA neurons and climbing defects (Fig. 4d-e, Additional file 14, Figure S4a-b). Dopaminergic overexpression of parkin or PINK1 has no effect on the proteosomal clearance of the mutant PARIS (Fig. 4f) and thus fails to attenuate the resultant toxic effects (Fig. 4d-e). These data taken together indicate that regulation of PARIS levels by PINK1 mediated phosphorylation and parkin dependent ubiquitination prevents PARIS induced neurotoxicity and promotes neuronal survival that is abolished in the absence of parkin or PINK1.

### PARIS expression leads to defects in mitochondrial biogenesis

**Fig. 5:**
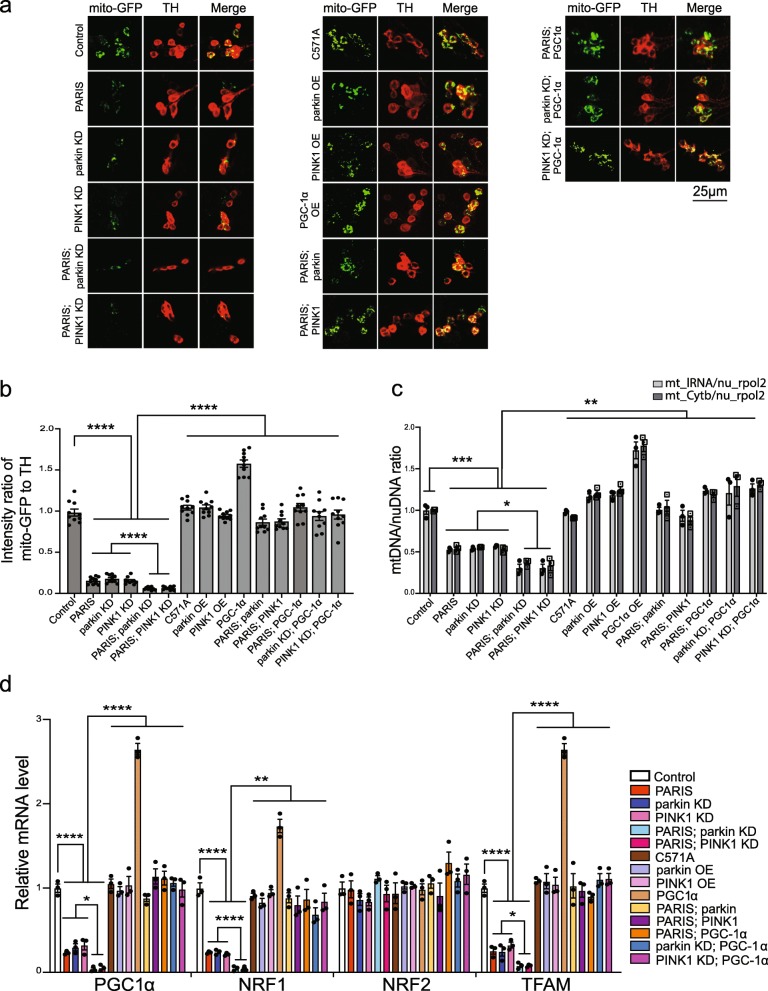
Rescue of PARIS induced dopaminergic mitochondrial biogenesis defects by PINK1 or parkin (**a**) Representative confocal maximum projections of mitochondria in DA neurons visualized by mito-GFP fluorescence (green), co-stained with anti-TH (red) in 20-day old flies in the genotypes indicated. Scale = 25 μM. **b** Quantification of mitochondria abundance within individual DA neurons using intensity ratio of mito-GFP to TH immunofluorescence shown. Th > mito-GFP flies served as control. *N* = 10 flies per indicated genotype. **c** Quantitative real-time PCR analysis of mitochondria DNA (mtDNA) relative to nuclear DNA (nuDNA) assessing mitochondrial DNA copy number in FACS sorted DA neurons in 20-day old flies in the genotypes indicated. Th > GFP flies served as control. Mean of three independent FACS experiments each employing 50 fly brains for the indicated genotypes depicted. **d** Quantitative RT-PCR analysis of transcript levels of *Drosophila* homologs of PGC-1α (Spargel), NRF1 (ewg), NRF-2 (Delg) and mitochondrial transcriptional factor A (TFAM) in FACS sorted DA neurons from 20-day old flies. Th > GFP flies served as control. Mean of three independent FACS experiments each using 50 fly brains per genotype shown. Quantitative data = mean ± SEM. One-way ANOVA *p < 0.05, **p < 0.01, *** *p* < 0.001, ****p < 0.0001. See also Additional file 14, Figure S4

PARIS represses expression of the transcriptional co-activator, PGC-1α which is a major regulator of mitochondrial biogenesis [13, 32, 34]. To examine if PARIS accumulation affected mitochondrial number and/or morphology, mitochondria in the DA neurons were labeled with mitochondrial targeted GFP (mito-GFP) to permit the detection and quantification of mitochondria. PARIS expression in DA neurons leads to a significant reduction in mito-GFP levels (Fig. 5a-b), indicative of a decrease in mitochondrial abundance. Unexpectedly, targeted knockdown of parkin or PINK1 in DA neurons also lead to a reduction in mito-GFP intensity that is further reduced in the setting of PARIS expression (Fig. 5a-b). Conversely, parkin or PINK1 overexpression in the PARIS flies restores the dopaminergic mito-GFP intensity to levels in control flies (TH > mito-GFP) (Fig. 5a-b). To verify if the observed decrease in mitochondrial abundance results from defects in mitochondrial biogenesis, mitochondrial DNA (mtDNA) abundance was assessed in DA neurons isolated by fluorescence activated cell sorting (FACS) using a dopaminergic GFP tag. The amount of mtDNA was assessed by the ratio of mtDNA to nuclear DNA (nuDNA) copy number, determined by quantitative real time PCR amplification of the mitochondrial cytochrome B (CytB) or the mitochondrial large ribosomal RNA (lRNA) gene [51] and the nuclear RNA polymerase 2 (rpol2) gene [52]. Compared to the control (TH > GFP), PARIS flies as well as the parkin or PINK1 knockdown flies exhibit a significant decrease in mtDNA copy number (Fig. 5c). Consistent with the reduction in mito-GFP intensity, PARIS in the setting of parkin or PINK1 knockdown further reduces the levels of mtDNA (Fig. 5c). Overexpression of parkin or PINK1 in PARIS flies however, restores mtDNA levels. (Fig. 5c). Overexpression of parkin or PINK1 by itself however did not significantly impact mitochondrial abundance or mtDNA levels in the DA neurons (Fig. 5a-c).

To determine a potential mechanism for the decrease in mitochondrial abundance observed under the different conditions, transcript levels of the *Drosophila* homologs of PGC-1α (Spargel) [53], NRF1 (ewg) [54], NRF2 (Delg) [55], and the NRF-1 target, mitochondrial transcription factor A (TFAM) [56] were examined specifically in DA neurons isolated by FACS. These genes were chosen as they regulate multiple aspects of the nucleo-mitochondrial interactions to promote mitochondrial biogenesis [57]. Quantitative RT-PCR measurements of these genes reveal substantial reduction of PGC-1α, NRF1 and TFAM mRNA levels in the DA neurons from PARIS flies but not in the C571A expressing DA neurons (Fig. 5d). DA neurons from the parkin or PINK1 knockdown flies also exhibit a similar decrease in transcript levels of PGC-1α, NRF1 and TFAM. NRF2 levels are however, unaffected under these conditions (Fig. 5d). While targeted knockdown of parkin or PINK1 in the PARIS flies exacerbates the repressive effect of PARIS on the above genes, overexpression of parkin or PINK1 ameliorates the effect and restores expression of the respective genes to levels observed in control flies (TH > GFP) (Fig. 5d). Overexpression of PGC-1α by itself in the DA neurons increases mitochondrial abundance through transcriptional upregulation of NRF1 and TFAM (Fig. 5a-d). Notably, overexpression of PGC-1α in the PARIS flies or in the setting of parkin or PINK1 knockdown restores mito-GFP levels (Fig. 5a-b) and also relieves the repressive effect on NRF1 and TFAM (Fig. 5d) which further substantiates PGC-1α as the downstream target of PARIS that mediates the observed biogenesis defects. Impingement of PGC-1α dependent transcriptional activity is also observable upon dopaminergic expression of the phosphodeficient PARIS DM. However, such mitotoxic effects mediated by the proteasomal resistant PARIS DM protein are however not ameliorated under conditions of parkin or PINK1 overexpression (Additional file 14, Figure S4c-f). Together, these data indicate that accumulation of PARIS under conditions of parkin or PINK1 deficiency leads to mitochondrial deficits through transcriptional repression of key factors that cooperatively regulate mitochondrial biogenesis.

Dopaminergic overexpression of PGC-1α in the PARIS and the parkin or PINK1 knockdown flies also prevents the loss of DA neurons and improves climbing performance (Fig. 3a-d, Additional file 9, Figure S2a-d; and Additional files 10-11, Tables S6-S7) indicating that impaired biogenesis affects dopaminergic neuronal viability and associated motor performance. Actin-Gal4 driven overexpression of human NRF1 as well as its fly counterpart (ewg) has lethal effect towards fly survival (Additional file 6, Table S4). Similarly, dopaminergic overexpression of ewg also caused 100% lethality (data not shown), which prevented further evaluation of the effect of NRF1 in the PARIS and parkin or PINK1 knockdown flies.

### *Drosophila* homolog of PARIS causes dopaminergic neurotoxicity that is attenuated by the PINK1/parkin pathway

Since dopaminergic knock down of PINK1 or parkin in *Drosophila* phenocopies the mitochondrial biogenesis defects observed under conditions of human PARIS accumulation, we were prompted to investigate if a *Drosophila* homolog of PARIS exists and further examine its dopaminergic effects. In this regard, a previously unannotated gene, CG15436 was recently reported to be a potential homolog of PARIS in *Drosophila* based on comparative homology modelling [58]. This putative *Drosophila* PARIS homolog (hereafter referred to as dPARIS) shares 12.8% identity and 20.5% similarity with human PARIS and contains a specialized N-terminal Zinc associated domain (ZAD) and C_2_H_2_ Zinc-finger double domains at its C-terminus. The ZAD domain is thought to be the *Drosophila* equivalent of KRAB domain that acts as a transcriptional repressor module in higher vertebrates and is also found in human PARIS. We therefore examined if dPARIS accumulation leads to dopaminergic neurotoxicity. Dopaminergic overexpression of dPARIS leads to loss of DA neurons in the major DA neuronal clusters PPL1, PPL2, PPM1/2, and PPM3 whereas dPARIS knockdown (KD) has no effect on neuronal survival (Additional file 15, Figure S5a-c). While the number of DA neurons in the dPARIS overexpressing flies is comparable to the control (TH-Gal4/+) and dPARIS KD flies on day 1 immediately after eclosion, a significant reduction in the number of neurons within all the dopaminergic clusters is notable on day 10 with a trend towards further reductions on day 30 and day 50 (Additional file 15, Figure S5a-c). Thus, dopaminergic accumulation of dPARIS leads to adult onset progressive loss of DA neurons similar to human PARIS. A concomitant age-dependent decline in climbing performance also accompanies the neuron loss under conditions of dPARIS overexpression but not its knockdown (Additional file 15, Figure S5d).

**Fig. 6:**
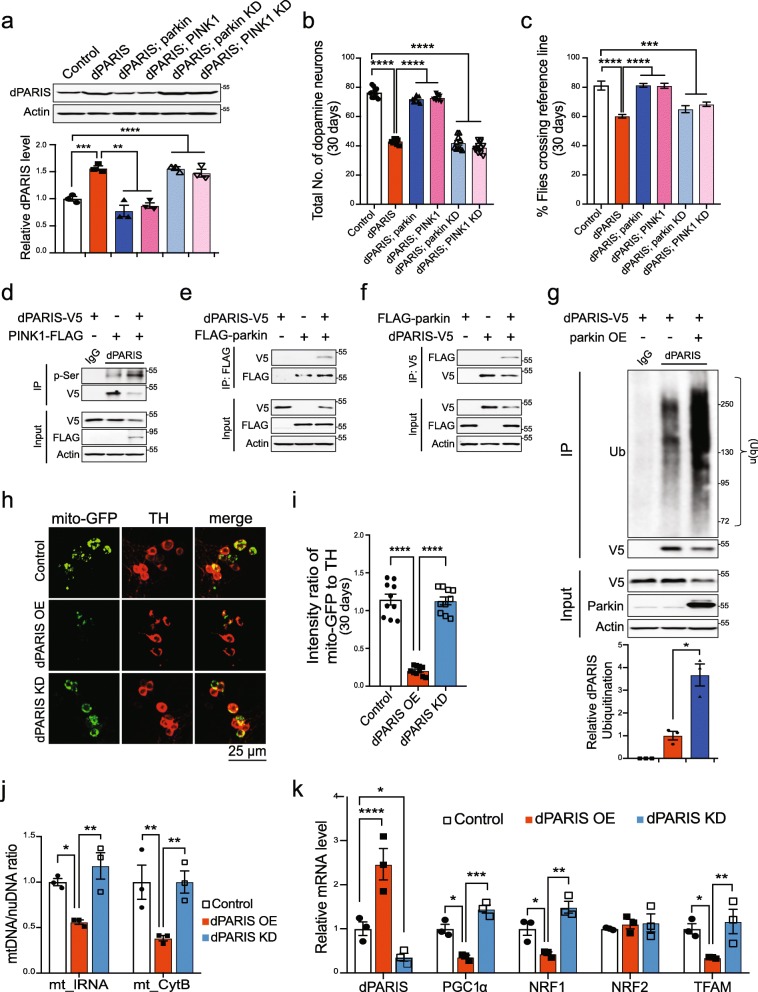
Dopaminergic neurotoxicity associated with *Drosophila* PARIS attenuated by PINK1/parkin pathway (**a**) Immunoblot analysis and quantification of *Drosophila* PARIS (dPARIS) in flies expressing the indicated transgenes under the control of TH-Gal4 driver, *N* = 3. **b** DA neuron counts in 30-day old flies. *N* = 10 flies per indicated genotype. **c** Climbing performance in 30-day old flies. *N* = 60 flies per indicated genotype. TH-Gal4/+ flies served as control. **d** Phosphorylation of dPARIS by PINK1 verified in *Drosophila S2* cells. Similar results observed in three independent experiments. **e** Coimmunoprecipitation using FLAG antibodies show interaction of C-terminal V5-tagged dPARIS with N-terminal FLAG tagged parkin in *Drosophila* S2 cells. *N* = 3. **f** Reciprocal coimmunoprecipitation using V5-antibodies verify parkin interaction with dPARIS in *Drosophila* S2 cells transfected with the indicated constructs, *N* = 3. **g** Ubiquitination of dPARIS assessed in *Drosophila* S2 cells transfected with the indicated constructs. Immunoblot analysis shows ubiquitination of dPARIS is enhanced by ectopic expression of parkin and leads to decreased dPARIS protein levels. Quantification of ubiquitinated dPARIS normalized to dPARIS-V5 protein levels shown, *N* = 3. **h** Representative maximal Z-projections of confocal stack images, immunostained for mito-GFP (green) and TH (red) in the indicated genotypes. Scale = 25 μM. **i** Quantification of intensity ratio of mito-GFP to TH immunofluorescence in the indicated genotypes. TH > mito-GFP flies served as control, *N* = 10 flies per genotype. **j** Mitochondrial DNA copy number assessed in FACS sorted DA neurons from 30-day old flies of the indicated genotypes. Mean ratio of mtDNA to nuDNA from three independent FACS experiments shown. TH > GFP flies used as control. **k** Quantitative RT-PCR analysis of *Drosophila* homologs of PARIS (dPARIS), PGC-1α (Spargel), NRF1 (ewg), NRF-2 (Delg) and mitochondrial transcriptional factor A (TFAM) in FACS sorted DA neurons from 30-day old flies. Th > GFP flies served as control. Mean of three independent FACS experiments per genotype shown. Quantitative data = mean ± SEM. One-way ANOVA *p < 0.05, **p < 0.01, *** *p* < 0.001, ****p < 0.0001. See also Additional file 15, Figure S5

We next examined if dPARIS is regulated via the PINK1/parkin pathway. Immunoblot analysis show that dopaminergic overexpression of parkin or PINK1 curbs dPARIS accumulation whereas knockdown of neither parkin or PINK1 has any discernible effect on dPARIS levels in the dPARIS overexpressing flies (Fig. 6a). DA neuron loss and climbing defects in the dPARIS overexpressing flies are also rescued by dopaminergic overexpression but not knockdown of parkin or PINK1 (Fig. 6b-c). These data identify dPARIS as a common substrate of the PINK1/parkin pathway.

### *Drosophila* PARIS is a substrate of PINK1/parkin pathway and causes mitochondrial biogenesis defects

We next sought to determine if dPARIS is a phosphorylation and ubiquitination substrate of PINK1 and parkin, respectively. Towards this end, the phosphorylation status of C-terminal V5-tagged dPARIS was monitored in *Drosophila* S2 cells in the presence of C-terminal FLAG tagged dPINK1. Following coimmunoprecipitation using a dPARIS specific antibody, immunoblot analysis of dPARIS using phosphoserine antibody shows increased phosphorylation of dPARIS under conditions of dPINK1 overexpression, indicating that dPARIS is a phosphosubstrate of PINK1 (Fig. 6d). Reciprocal coimmunoprecipitation experiments indicate that dPARIS interacts with parkin (Fig. 6e and f). Coimmunoprecipitation using a dPARIS specific antibody show increased ubiquitination of dPARIS under conditions of parkin overexpression. Increased dPARIS ubiquitination is observable in the form of a smear, characteristic of polyubiquitinated proteins and leads to substantially reduced cellular levels of dPARIS (Fig. 6g). This indicates that parkin mediated ubiquitination of dPARIS targets it for proteosomal degradation, thereby promoting DA neuron survival in vivo.

In order to uncover the mechanism underlying dPARIS neurotoxicity, we next examined the effects of dPARIS accumulation on mitochondrial abundance in the DA neurons. Quantification of mitochondria labeled within the DA neurons using mitochondrial targeted GFP fluorescence (mito-GFP) reveals marked reduction in mitochondrial numbers under conditions of dopaminergic overexpression of dPARIS whereas dPARIS knockdown has no discernible effect on mitochondrial abundance (Fig. 6h and i). To further verify this observation, mtDNA copy number was assessed in FACS sorted DA neurons by measuring the mtDNA/nuDNA ratio of two mitochondrial encoded genes (mt_lRNA and mt_CytB) using quantitative real time PCR amplification. These studies also reveal a significant decrease in mtDNA copy number in the dPARIS overexpressing flies (Fig. 6j). Quantitative RT-PCR measurements of dopaminergic mRNA levels of the *Drosophila* homologs of PGC-1α (Spargel), NRF-1 (ewg) and mitochondrial transcription factor (TFAM) also show marked repression of these genes under conditions of dPARIS overexpression which did not impact NRF2 (Delg) expression (Fig. 6k). Dopaminergic knockdown of dPARIS however, has no effect on the transcript level of the above biogenesis promoting factors (Fig. 6k) and as such mtDNA copy numbers in the dPARIS KD flies are comparable to the control flies (TH > GFP) (Fig. 6j).

**Fig. 7:**
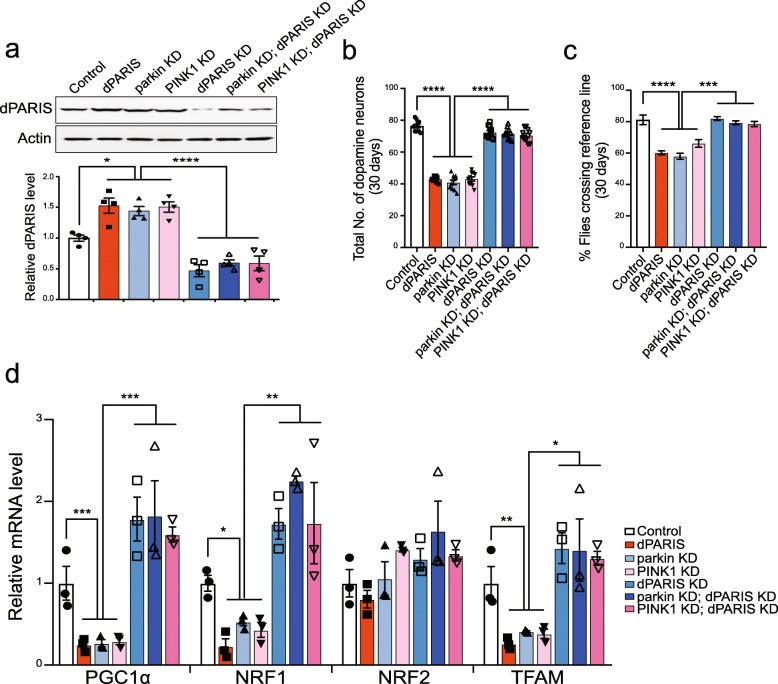
Dopaminergic knockdown of *Drosophila* PARIS rescues dopaminergic neurotoxicity associated with reduced parkin or PINK1 activity. **a** Immunoblot analysis and quantification of dPARIS protein levels in flies expressing the indicated transgenes under the control of TH-Gal4 driver. **b** Dopaminergic knockdown of dPARIS promotes DA neuron survival under conditions of parkin or PINK1 knockdown in 30-day old flies. *N* = 10 flies per genotype. **c** Climbing defects associated with dopaminergic knockdown of parkin or PINK1 restored by dPARIS knockdown in DA neurons. *N* = 60 flies per indicated genotype. TH-Gal4/+ flies served as control. **d** Quantitative RT-PCR analysis of *Drosophila* homologs of PGC-1α (Spargel), NRF1 (ewg), NRF-2 (Delg) and mitochondrial transcriptional factor A (TFAM) in 30-day old fly heads from the indicated genotypes, *N* = 3. Quantitative data = mean ± SEM. One-way ANOVA *p < 0.05, **p < 0.01, *** *p* < 0.001, ****p < 0.0001. See also Additional file 16, Figure S6

Notably, dopaminergic knockdown of dPARIS promotes DA neuron survival (Fig. 7a-b, Additional file 16, Figure S6a-b) and improves climbing performance under conditions of parkin or PINK1 knockdown (Fig. 7c), further verifying that PINK1/parkin regulation is specific to dPARIS in the DA neurons. Of note, dopaminergic knockdown of dPARIS in the setting of parkin or PINK1 knockdown relieves transcriptional repression of PGC-1α, NRF-1 and TFAM and restores the respective transcripts to control levels (Fig. 7d). Together, these data indicate that dPARIS accumulation exerts neurotoxic effects similar to human PARIS and causes mitochondrial biogenesis defects through transcriptional repression of the *Drosophila* homolog of PGC-1α and its downstream transcription factors NRF-1 and TFAM that is detrimental to DA neuron survival.

## Discussion

The interplay between PINK1 and parkin and the identification of their common mitochondrial substrates exemplify the mitochondrial axis of PD pathogenesis [4, 22]. Nevertheless, which of the PINK1/parkin regulated mitochondrial pathways assume a pathogenic role when altered and drive dopaminergic degeneration in PD is presently unclear. We show here that defects in mitochondrial biogenesis underlie age-related progressive loss of DA neurons and climbing deficits in *Drosophila* under conditions of PINK1 or parkin insufficiency. These dopaminergic phenotypes are consistent with those caused by hPARIS overexpression in *Drosophila* and stem from defects in mitochondrial biogenesis that are PGC-1α dependent. Coordinated phosphorylation and ubiquitination by PINK1 and parkin, respectively regulate cellular levels of hPARIS [13, 32]. Our studies indicate that the single *Drosophila* homolog of PARIS (dPARIS) is similarly subject to proteosomal regulation by the PINK1/parkin pathway. This is further substantiated by our findings that dopaminergic overexpression of PINK1 or parkin effectively attenuates the degenerative phenotypes associated with dPARIS accumulation. Notably, dopaminergic knockdown of dPARIS is sufficient to prevent the mitochondrial biogenesis defects, DA neuron loss and locomotor deficits under conditions of reduced PINK1 or parkin activity. Together, our studies highlight the evolutionarily conserved nature of PINK1/parkin regulation of PARIS and its critical requirement in sustaining mitochondrial quality control in the DA neurons via the PARIS/PGC-1α axis.

Analogous to the KRAB domain in mammalian Zinc-finger proteins, the ZAD domain in *Drosophila* mediates transcriptional repression [59, 60]. This is evident in the case of dPARIS as well which, similar to its human counterpart, negatively impacts transcription of the *Drosophila* homolog of PGC-1α and other key mediators of mitochondrial biogenesis in fly DA neurons. Thus, PARIS regulation by the PINK1/parkin pathway and its transcriptional regulatory function is evolutionarily conserved. PGC-1α is a transcriptional co-activator that localizes to the nucleus and regulates expression of a myriad of genes with mitochondrial roles including mitochondrial biogenesis and respiration [33]. PGC-1α repression by PARIS impacts expression of such genes leading to an overall decrease in mitochondrial content and function. In support of this notion, we observe a marked decrease in mitochondrial DNA (mtDNA) content in the DA neurons under conditions of PARIS accumulation (human or *Drosophila*) coupled with decreased expression of the transcription factor NRF1 that activates transcription of a number of genes involved in respiratory function [61]. Furthermore, the nucleus encoded NRF1 target gene, TFAM that translocates to the mitochondria and drives mitochondrial DNA replication and transcription [62, 63] also exhibits decreased expression. Notably, the transcript level of these genes is unaffected by the transcriptionally inactive hPARIS mutant C571A. The repressive effect is also relieved under conditions of dPARIS knockdown. By reducing the amount of TFAM in the mitochondria, PARIS accumulation could in effect impact the rate of transcription and abundance of respiratory subunits, rRNAs and tRNAs encoded in the mitochondrial genome. Since transcripts from the light strand promoter of mtDNA prime replication of the heavy strand DNA [64], the rate of transcription as determined by TFAM abundance may in turn affect copy number of mtDNA as is evident when PARIS is overexpressed. Thus, it is conceivable that by transcriptionally modulating the amount and function of these two key regulators of mitochondrial biogenesis, PARIS mediated repression of PGC-1α sets in motion a cascade of events that ultimately impinge on mitochondrial homeostasis. *Drosophila* homologs of PGC-1α, NRF1 and TFAM were also represented in a genome-wide RNAi screen in *Drosophila* S2 cells among genes that function as positive regulators of mtDNA maintenance [65], further implying that the decreased mitochondrial content we observe in the PARIS flies results from PARIS mediated repression of these factors. PGC-1α potently induces and co-activates both NRF1 and NRF2 function [66]. However, the lack of any discernible effect on dopaminergic NRF2 levels under conditions of PARIS accumulation suggests that modulation of mitochondrial content and function in the DA neurons is primarily mediated via the PGC-1α /NRF1 pathway. Our data also strongly suggests that defects in mitochondrial biogenesis play a causative role in the loss of DA neurons as overexpression of *Drosophila* PGC-1α (Spargel) not only relieves the repressive effect on NRF1 and TFAM to restore mitochondrial content but also prevents the age-related dopaminergic cell death observed in flies overexpressing hPARIS.

*Drosophila* PINK1 and parkin null mutants exhibit mild DA neuron loss in some [16, 19, 20], but not all studies [14, 15, 18]. However, we observe a substantial and significant loss of DA neurons in all of the dopaminergic clusters with ~ 60% reduction of PINK1 or parkin expression upon induction of the respective shRNA response in DA neurons. Our observations therefore suggest that PINK1 and parkin may have cell-type specific regulatory roles that renders dopaminergic neurons particularly susceptible to inactivation of these proteins. In the complete absence of PINK1 or parkin activity as is the case in the null flies, it is likely that such cell type specific physiological requirements are masked by activation of other developmental compensatory mechanisms. The unique physiological characteristics of the substantia nigra DA neurons, such as their continuous pacemaking activity, calcium buffering requirements, complex arborization and high number of neurotransmitter release sites are likely to impose disproportionately high energy demands on these neurons making them particularly vulnerable to mitochondrial dysfunctions [67]. Certain electrophysiological properties of mammalian DA neurons show evolutionary conservation in flies. For instance, the hyperpolarization-activated cation current, I_h_, that is known to contribute to the autonomous pacemaking activity in mammalian DA neurons [68] also maintains DA signaling and locomotor activity in *Drosophila* [39]. These features could explain why dopaminergic neurons display enhanced susceptibility to PARIS neurotoxicity in our fly models while histaminergic and cholinergic neurons are spared.

Loss of *Drosophila* PINK1 or parkin has also been shown to affect mitochondrial integrity [14, 18–20], an effect widely held to be causal to the degeneration of DA neurons. However, such mitochondrial defects in these studies are evident in only a subset of DA neurons and also persist in diverse other cell types with high energy demands implying that additional PINK1/parkin dependent mitochondrial pathways may be at play in the DA neurons. In fact, we observe that dopaminergic downregulation of parkin or PINK1 leads to a marked decrease in dopaminergic mitochondrial abundance. Accompanying this mitochondrial defect is the decrease in *Drosophila* PGC-1α, NRF1 and TFAM transcript levels. While these effects are reminiscent of that observed in the PARIS flies, remarkably, they are all rescued by overexpression of *Drosophila* PGC-1α implying that the PINK1/parkin pathway facilitates PGC-1α dependent transcriptional events that promote mitochondrial biogenesis. Regulation of PARIS appears to be a point of convergence of the PINK1/parkin pathway with factors that co-operatively promote the assembly of functional mitochondria. This is further supported by genetic suppression of the biogenesis defects and neuronal loss in the PARIS (human or *Drosophila*) overexpressing flies by PINK1 or parkin overexpression. It is therefore likely that under conditions of PINK1 or parkin knockdown, residual activity of these proteins while adequate to maintain the dynamic balance between fission and fusion events is albeit insufficient to prevent PARIS from accumulating and negatively impacting biogenesis.

PINK1 and parkin have been widely suggested to orchestrate the autophagic removal of dysfunctional mitochondria (mitophagy) in cultured mammalian cells [69, 70] and *Drosophila* cell lines [71]. Since PINK1 and parkin genetically interact with components of the mitochondrial fission and fusion machinery [72], regulated fission/fusion events in addition to sustaining a functional pool of mitochondria are thought to help ‘sort’ terminally damaged mitochondria for degradation [71]. Consequently, defective mitophagy and/or mitochondrial dynamics have taken center stage as putative pathophysiological mechanisms in PD. The in vivo significance of other PINK1/parkin co-substrates, genetic/molecular interactors of either PINK1 or parkin, and modifiers of the PINK1/parkin mitochondrial functions have been explored by other studies in the *Drosophila* PINK1 or parkin null mutants [71, 73–81]. These studies have for the most part relied on the restoration of anatomical and locomotor deficiencies in PINK1 or parkin mutants that are presumed to result from defects in mitochondrial fission and thereby mitophagy in other energy demanding tissues besides the DA neurons. However, as noted above, because of the inconsistent effects observed with DA neuronal loss in the PINK1 or parkin null flies, it is not feasible to establish a compelling link between these mitochondrial pathways and neurodegeneration in these models. Additionally, genetic manipulations that rectify mitochondrial dynamics in the absence of PINK1 or parkin [72] fail to completely restore bioenergetic defects in the PINK1 null flies [82, 83], suggesting that alterations to mitochondrial morphology per se are not sufficient to prevent DA neurodegeneration in vivo under conditions of PINK1 or parkin inactivation. Moreover, as reviewed in the introduction, direct evidence indicative of defects in mitophagy specifically in the DA neurons of *Drosophila* or any in vivo PD model is not available to date. Studies using the recently developed mitophagy reporter mice and *Drosophila* also show that mitophagy in vivo is rather constitutive, exhibits spatiotemporal variability and is minimally impacted by loss of PINK1 or parkin [25, 27]. It therefore appears that a certain mitophagy threshold exists to maintain mitochondrial network integrity in vivo. Perhaps, as yet unidentified intrinsic and extrinsic signals serve as ‘second-hits’ in the absence of PINK1 or parkin activity to tip such a mitophagy threshold. Delineation of such signals could be instructive in realizing the pathophysiological relevance of mitophagy in the context of PD. Moreover, if defects in mitophagy were driving the mitochondrial or dopaminergic deficits in PD, one might expect an increase in mitochondrial mass due to inability to clear damaged mitochondria. In contrast to this notion, mass spectrometry in the heads of PINK1 and parkin KO files suggest that mitochondrial turnover might be separate from mitophagy [84]. Moreover, in the midbrain of germline parkin KO mice, mass spectrometry revealed decreased mitochondrial proteins [85] in the setting of reduced respiratory capacity [86], consistent with findings reported here. There was also no accumulation of mitochondria in PINK1 KO mice [87]. DA neurodegeneration and mitochondrial dysfunction in germline parkin KO mice crossed to the DNA polymerase subunit gamma (POLG) 257A mitochondrial mutator mouse does not seem to be due to defects in mitophagy since there was no increase in mitochondrial mass, despite evidence of elevated phospho-ubiquitin, a marker of PINK1 activation and mitophagy [22]. Crossing the germline parkin KO to the PD-mito-PstI mouse also led to enhanced DA neuron degeneration in the setting of mitochondrial DNA damage without an increase in mitochondrial mass or mitophagy [88]. Genetic perturbation of germline parkin KO mice by crossing to mitochondrial transcription factor A (TFAM) KO mice failed to exhibit indices of impaired mitophagy [89]. Thus, although the absence of parkin or PINK1 leads to defects in mitophagy, it is likely that other aspects of parkin/PINK1 function predominate and drive the alterations in mitochondrial function and the ultimate loss of DA neurons [23, 90].

In adult conditional PINK1 or parkin knockdown mice that have age-dependent progressive loss of DA neurons [13, 32], there is a reduction in mitochondrial mass with no observable defects in mitophagy, consistent with a mitochondrial biogenesis defect [34]. In this regard, the widespread dopaminergic neurodegeneration we observe under conditions of diminished PINK1 and parkin activity and the fact that they can be effectively rescued by ameliorating the biogenesis defects indicates that the PINK1/parkin pathway mediated regulation of mitochondrial biogenesis may have more fundamental roles in sustaining optimal mitochondrial function in the DA neurons. We cannot exclude the possibility that there is convergence and interplay of mitophagy and mitochondrial biogenesis in the loss of DA neurons due to PINK1 or parkin loss. Future studies will be required to investigate the relationship mitophagy and mitochondrial biogenesis in the loss of DA neurons in PD.

Notably, to our knowledge, PARIS is the only cosubstrate of PINK1 and parkin to specifically accumulate in the DA neurons and cause neurodegeneration and locomotor defects that stem from disrupted DA signaling. Not only does biogenesis augment synthesis of mitochondrial proteins to meet cellular energy demands but it also serves to replace depleted parts. It is therefore conceivable that when the PINK1/parkin pathway eliminates dysfunctional mitochondria via mitophagy, it also promotes proteosomal degradation of PARIS as a homeostatic mechanism to counterbalance the effect and replenish a healthy pool of mitochondria through PGC-1α/NRF1 mediated mitochondrial biogenesis. Loss of PINK1 in *Drosophila* causes deficiency of complex I and complex IV dependent respiration [82]. Reduced complex I function has also been observed in PD patients with parkin mutations [91], in induced pluripotent stem (iPS) cells derived for PD patients with PINK1 mutations [92] as well as in PINK1 and parkin knockout mice [87, 93]. These observations suggest that PINK1 and parkin are needed to maintain complex I function and electron transport chain activity under steady state conditions. Since the downstream targets of PGC-1α and NRF1 include a wide range of nuclear genes coding for all five respiratory complex (RC) subunits, impaired synthesis and/or assembly of RC components due to defective biogenesis could have inhibitory feed forward effects that ultimately impinge on mitochondrial energy metabolism.

## Conclusions

By employing *Drosophila* models, the current study provides further validation that defective mitochondrial biogenesis drives dopaminergic neurotoxicity under conditions of PINK1/parkin insufficiency. These findings add yet another dimension to the mitochondrial quality control process spearheaded by the PINK1/parkin pathway.

The *Drosophila* models of PARIS accumulation described in this study recapitulate the cardinal neurodegenerative features of PD and thus could facilitate the identification of novel physiologically relevant regulators with tremendous therapeutic potential.

## Supplementary information

**Additional file 1: Table S1.** List of *Drosophila melanogaster* lines used in this study.

**Additional file 2: Table S2.** List of primers used in this study.

**Additional file 3: Table S3.** Summary of changes in life span and statistical analysis in adult flies ubiquitously expressing wild type or mutant PARIS.

**Additional file 4: Video-1.** Climbing performance of Control, PARIS and C571A flies. Ubiquitous expression of PARIS leads to marked decline in climbing performance compared to control (Act/+) and C571A flies (MP4).

**Additional file 6: Table S4.** Genetic rescue of PARIS lethality.

**Additional file 7: Table S5.** Summary of observed versus expected number of TH-Gal4; parkin null transheterozygotes.

**Additional file 8: Figure S1.** Ubiquitous expression of PARIS in *Drosophila* does not affect 5-HT neurons or cause muscle degeneration.

**Additional file 9: Figure S2.** PARIS induced dopaminergic neurodegeneration rescued by PINK1 and parkin.

**Additional file 10: Table S6.** Statistical comparison of total number of DA neurons and climbing performance by genotypes.

**Additional file 11: Table S7.** Statistical comparison of DA neuron number and climbing performance by age groups.

**Additional file 13 Figure S3.** Specific expression of PARIS in *Drosophila* 5-HT or cholinergic neurons does not cause neuron loss and climbing defects.

**Additional file 14: Figure S4.** Dopaminergic overexpression of parkin or PINK1 does not abrogate neurotoxicity associated with PARIS phosphomutant.

**Additional file 15: Figure S5.***Drosophila* homolog of PARIS causes age-dependent loss of DA neurons and climbing defects.

**Additional file 16: Figure S6.** Dopaminergic neurotoxicity resulting from reduced parkin or PINK1 activity prevented under conditions of dPARIS knockdown.

## Data Availability

The datasets used and/or analyzed during the current study are included in this article and its supplementary information files. The datasets are available from the corresponding author on reasonable request.

## Abbreviations

Cha: Choline Acetyltransferase
CLEM: Correlative Light and Electron Microscopy
CytB: Cytochrome B
DA: Dopamine
FACS: Fluorescence Activated Cell Sorting
GFP: Green Fluorescent Protein
KD: Knockdown
KO: Knockout
KRAB: Krüppel Associated Box
lRNA: large ribosomal RNA
Mef2: Myocyte Enhancer Factor-2
mtDNA: Mitochondrial DNA
NRF1: Nuclear respiratory factor 1
NRF2: Nuclear respiratory factor s
nuDNA: Nuclear DNA
PARIS: Parkin Interacting Substrate
PD: Parkinson’s disease
PGC-1α: Peroxisome Proliferator-Activated Receptor Gamma Co-activator 1-alpha
PINK1: PTEN-induced kinase 1
PM: Protocerebral Posterior Medial
PPL: Protocerebral Posterior Lateral
RC: Respiratory complex
rpol2: RNA polymerase 2
TFAM: Mitochondrial Transcription Factor
Trh: Tryptophan Hydroxylase
ZAD: Zinc Associated Domain
