# Supplementary material for: PARIS induced defects in mitochondrial biogenesis drive dopamine neuron loss under conditions of parkin or PINK1 deficiency
Source: Mol Neurodegener. 2020 Mar 5;15:17. doi: 10.1186/s13024-020-00363-x (PMC7057660; doi:10.1186/s13024-020-00363-x)
Supplement: Supplementary file 3 — Additional file 1: Table S1. List of Drosophila melanogaster lines used in this study. [file 13024_2020_363_MOESM1_ESM.docx]

**ADDITIONAL FILE 1:**

**Table S1. List of *Drosophila melanogaster* lines used in this study.**

| **Genotype** | **Source** | **Identifier** |
| --- | --- | --- |
| *D. melanogaster* W1118 | BDSC | Cat# 3605, RRID:BDSC_3605 |
| *D. melanogaster* UAS-dPARIS (CG15436) | FlyORF | Cat# F000677, RRID:FlyBase_FBst0501132 |
| *D. melanogaster* UAS-dPARIS shRNA (CG15436) | VDRC | Cat# v39986, RRID:FlyBase_FBst0463329 |
| *D. melanogaster* UAS-parkin shRNA | BDSC | Cat# 37509, RRID:BDSC_37509 |
| *D. melanogaster* UAS-PINK1 shRNA | BDSC | Cat# 38262, RRID:BDSC_38262 |
| *D. melanogaster* UAS-parkin WT | BDSC | Cat# 51651, RRID:BDSC_51651 |
| *D. melanogaster* UAS-PINK1 WT-3 | BDSC | Cat# 51648, RRID:BDSC_51648 |
| *D. melanogaster* UAS-GFP | BDSC | Cat# 6874, RRID:BDSC_6874 |
| *D. melanogaster* UAS-mito-GFP | BDSC | Cat# 8442, RRID:BDSC_8442 |
| *D. melanogaster* UAS-EGFP shRNA | BDSC | Cat# 41556, RRID:BDSC_41556 |
| *D. melanogaster* Actin-Gal4 | BDSC | Cat# 4414, RRID:BDSC_4414 |
| *D. melanogaster* TH-Gal4 | BDSC | Cat# 8848, RRID:BDSC_8848 |
| *D. melanogaster* Mef2-Gal4 | BDSC | Cat# 27390, RRID:BDSC_27390 |
| *D. melanogaster* Trh-Gal4 | BDSC | Cat# 38388, RRID:BDSC_38388 |
| *D. melanogaster* Cha-Gal4 | BDSC | Cat# 6793, RRID:BDSC_6793 |
| *D. melanogaster* D42-Gal4 | BDSC | Cat# 8816, RRID:BDSC_8816 |
| *D. melanogaster* parkin25 | Dr. Jongkyeong Chung, Seoul National University | NA |
| *D. melanogaster* PINK1 B9 | Dr. Jongkyeong Chung, Seoul National University | NA |
| *D. melanogaster* UAS-PINK1 WT-2 | Dr. Leo Pallanck, Univ. of Washington | NA |
| *D. melanogaster* UAS-Spargel (Srl)-2 | Dr. Christian Frei, Univ. of Zurich | NA |
| *D. melanogaster* UAS-Spargel (Srl)-3 | Dr. David Walker, UCLA | NA |
| *D. melanogaster* UAS-NRF-V5 | Dr. Yashi Ahmed, Dartmouth College | NA |
| *D. melanogaster* UAS-PARIS-1 | This paper | NA |
| *D. melanogaster* UAS-PARIS-2 | This paper | NA |
| *D. melanogaster* UAS-PARIS-3 | This paper | NA |
| *D. melanogaster* UAS-C571A-1 | This paper | NA |
| *D. melanogaster* UAS-C571A-2 | This paper | NA |
| *D. melanogaster* UAS-PARIS DM2 | This paper | NA |
| *D. melanogaster* UAS-PARIS DM3 | This paper | NA |
| *D. melanogaster* UAS-parkin shRNA; UAS-PARIS | This paper | NA |
| *D. melanogaster* UAS-PINK1 shRNA; UAS-PARIS | This paper | NA |
| *D. melanogaster* UAS-parkin WT; UAS-PARIS | This paper | NA |
| *D. melanogaster* UAS-PARIS; UAS-PINK1 WT | This paper | NA |
| *D. melanogaster* UAS-Srl; UAS-PARIS | This paper | NA |
| *D. melanogaster* UAS-parkin shRNA; UAS-Srl | This paper | NA |
| *D. melanogaster* UAS-PINK1 shRNA; UAS-Srl | This paper | NA |
| *D. melanogaster* UAS-parkin WT; UAS-PARIS DM3 | This paper | NA |
| *D. melanogaster* UAS-PARIS DM2; UAS-PINK1 WT | This paper | NA |
| *D. melanogaster* UAS-parkin shRNA; UAS-dPARIS | This paper | NA |
| *D. melanogaster* UAS-PINK1 shRNA; UAS-dPARIS | This paper | NA |
| *D. melanogaster* UAS-parkin WT; UAS-dPARIS | This paper | NA |
| *D. melanogaster* UAS-PINK1 WT-2; UAS-dPARIS | This paper | NA |
| *D. melanogaster* UAS-parkin shRNA; TH-Gal4 | This paper | NA |
| *D. melanogaster* UAS-PINK1 shRNA; TH-Gal4 | This paper | NA |
| *D. melanogaster* UAS-GFP; TH-Gal4 | This paper | NA |
| *D. melanogaster* UAS-mito-GFP; TH-Gal4 | This paper | NA |
