# Supplementary material for: PARIS induced defects in mitochondrial biogenesis drive dopamine neuron loss under conditions of parkin or PINK1 deficiency
Source: Mol Neurodegener. 2020 Mar 5;15:17. doi: 10.1186/s13024-020-00363-x (PMC7057660; doi:10.1186/s13024-020-00363-x)
Supplement: Supplementary file 4 — Additional file 2: Table S2. List of primers used in this study. [file 13024_2020_363_MOESM2_ESM.docx]

**ADDITIONAL FILE 2:**

**Table S2. List of primers used in this study.**

| **Primer ID** | **Sequence (5’-3’)** | **Source** |
| --- | --- | --- |
| **Primers for dPARIS and dparkin cloning** |  |  |
| dPARIS Fwd | TAAGCAGGTACCATGGCGGAAATATGCCGAGTTTG | This paper |
| dPARIS Rev | TGCTTAGATATC CTTGTGTATCTCGAGGATATGTC | This paper |
| dparkin-FLAG-Fwd | TAAGCACTCGAGCACCATGGACTACAAAGACG -  ATGACGACAAGATGAGTTTTATTTTTAAATTTATTG | This paper |
| dparkin-Fwd | TAAGCACTCGAGCACCATGAGTTTTATTTTTA AATTTATTG | This paper |
| dparkin-Rev | TGCTTACCGCGGTTAGCCGAACCAGTGGGC | This paper |
| **Primers for quantitative RT-PCR** |  |  |
| Spargel-Fwd | TGTCACACAGATCTCGCTCA | This paper |
| Spargel-Rev | AACCGCCGTTTCCAATACTG | This paper |
| Ewg-Fwd | CGAACTTCCTGGCTTGGTAAT | This paper |
| Ewg-Rev | CTTCCCTCGTCCCGTAGAATA | This paper |
| Delg-Fwd | AAGAGTTCCTCCAGCGAAAG | This paper |
| Delg-Rev | AGACTGGCTTGACATCCTTG | This paper |
| TFAM-Fwd | CTGTCTAAGAACTGGTCCGATG | This paper |
| TFAM-Rev | TACTTTGTTCGCTCCTCCAC | This paper |
| RP49-Fwd | CCAAGATCGTGAAGAAGCG | This paper |
| RP49-Rev | GTTGTCGATACCCTTGGGC | This paper |
| dparkin-Fwd | TTGGGCGAGCGTCAGTTTAT | This paper |
| dparkin-Rev | GCTTGAAGTGATGAATCTCCTCG | This paper |
| dPINK1-Fwd | TGCCCATCGTGACCTAAAGT | This paper |
| dPINK1-Rev | GCCAGACAACAGCCAAAGTC | This paper |
| dPARIS-Fwd | ATGGCGGAAATATGCCGAG | This paper |
| dPARIS-Rev | CGCGATCATCTCGGCAATGG | This paper |
| **Primers for measurement of mtDNA density** |  |  |
| mt_lRNA-Fwd | AGTCTAACCTGCCCACTGAA | This paper |
| mt_lRNA-Rev | CCAACCATTCATTCCAGCCT | This paper |
| mt_CytB-Fwd | CCTTTAGTAACACCTGCCCATA | This paper |
| mt_CytB-Rev | AACTGGTCGAGCTCCAATTC | This paper |
| rpol2-Fwd | AGGCGTTTGAGTGGTTGG | Correa et al., 2012 |
| rpol2-Rev | TGGAAGGTGTTCAGTGTCATC |  |
