# Supplementary material for: PARIS induced defects in mitochondrial biogenesis drive dopamine neuron loss under conditions of parkin or PINK1 deficiency
Source: Mol Neurodegener. 2020 Mar 5;15:17. doi: 10.1186/s13024-020-00363-x (PMC7057660; doi:10.1186/s13024-020-00363-x)
Supplement: Supplementary file 5 — Additional file 3: Table S3. Summary of changes in life span and statistical analysis in adult flies ubiquitously expressing wild type or mutant PARIS. [file 13024_2020_363_MOESM3_ESM.docx]

ADDITIONAL FILE 3:

Table S3. Summary of changes in life span and statistical analysis in adult flies ubiquitously expressing wild type or mutant PARIS.

| **Genotype** | **Median LS*** | **n** | **Log-Rank** | |
| --- | --- | --- | --- | --- |
|  |  |  | **Chi-Square** | **P-value** |
| Control vs PARIS-1 | 27 | 100 | 200.8 | <0.0001 |
| Control vs PARIS-2 | 30 | 100 | 191.1 | <0.0001 |
| Control vs PARIS-3 | 30 | 100 | 194.6 | <0.0001 |
| Control vs C571A-1 | 48 | 100 | 50.89 | <0.0001 |
| Control vs C571A-2 | 54 | 100 | 48.26 | <0.0001 |
| PARIS-1 vs PARIS-1+L-DOPA | 42 | 100 | 64.13 | <0.0001 |
| PARIS-2 vs PARIS-2+L-DOPA | 45 | 100 | 57.19 | <0.0001 |
| PARIS-3 vs PARIS-3+L-DOPA | 48 | 100 | 79.83 | <0.0001 |

*LS indicates life span and n indicates number of adult flies analyzed. Data analyzed using Log-rank (Mantel-Cox) test and show statistically significant reduction in lifespan in the three independent PARIS fly lines. Median LS in control = 60 days.
