# Supplementary material for: PARIS induced defects in mitochondrial biogenesis drive dopamine neuron loss under conditions of parkin or PINK1 deficiency
Source: Mol Neurodegener. 2020 Mar 5;15:17. doi: 10.1186/s13024-020-00363-x (PMC7057660; doi:10.1186/s13024-020-00363-x)
Supplement: Supplementary file 7 — Additional file 6: Table S4. Genetic rescue of PARIS lethality. [file 13024_2020_363_MOESM6_ESM.docx]

ADDITIONAL FILE 6:

Table S4. Genetic rescue of PARIS lethality.

| **Genotype** | **Actin-Gal4** | **Cyo** | **Total** | **% with Actin-Gal4** | **% Expected versus w1118** |
| --- | --- | --- | --- | --- | --- |
| w1118 | 98 | 107 | 205 | 47.80 | 100.00 |
| UAS.PARIS | 27 | 200 | 227 | 11.89 | 24.88 |
| UAS.C571A | 89 | 104 | 193 | 46.11 | 96.46 |
| UAS.hparkin | 116 | 139 | 255 | 45.49 | 95.16 |
| UAS. hparkin + PARIS | 42 | 175 | 217 | 19.35 | 40.49 |
| UAS.dparkin | 121 | 138 | 259 | 46.72 | 97.73 |
| UAS.dparkin + PARIS | 38 | 161 | 199 | 19.10 | 39.94 |
| UAS.hPink1 | 0 | 220 | 220 | 0.00 | 0.00 |
| UAS.hPink1 + PARIS | 0 | 212 | 212 | 0.00 | 0.00 |
| UAS.dPink1 | 0 | 174 | 174 | 0.00 | 0.00 |
| UAS.dPink1 + PARIS | 0 | 185 | 185 | 0.00 | 0.00 |
| UAS.PGC1α | 0 | 166 | 166 | 0.00 | 0.00 |
| UAS.PGC1α + PARIS | 0 | 158 | 158 | 0.00 | 0.00 |
| UAS.NRF1 | 0 | 175 | 175 | 0.00 | 0.00 |
| UAS.NRF1 + PARIS | 0 | 203 | 203 | 0.00 | 0.00 |
| UAS.EWG | 0 | 196 | 196 | 0.00 | 0.00 |
| UAS.EWG + PARIS | 0 | 186 | 186 | 0.00 | 0.00 |
| parkin KO | 21 | 25 | 46 | 45.65 | 95.50 |
| parkin KO + UAS.PARIS | 0 | 57 | 57 | 0.00 | 0.00 |
| Pink1 KO | 20 | 31 | 51 | 39.22 | 82.03 |
| Pink1 KO + UAS. PARIS | 0 | 48 | 48 | 0.00 | 0.00 |

‘Actin-Gal4’ shows the number of flies eclosed with Actin-Gal4 driver, ‘*Cyo*’ refers to number of flies eclosed with *Cyo* balancer. ‘Total’ shows the total number of F1 flies eclosed in each cross. ‘% With Actin-Gal4’ shows the number of flies eclosed with Actin-Gal4 driver divided by the total number of flies eclosed. The 47.80% eclosed flies (without *Cyo*) from the control cross (Act-Gal4/*Cyo* x *W^1118^*) were set as 100% eclosed. ‘% Expected versus *W^1118^* ‘shows the percentage of Actin-Gal4 expressing flies (non-*Cyo*) eclosed divided by 47.80%.
