# Supplementary material for: PARIS induced defects in mitochondrial biogenesis drive dopamine neuron loss under conditions of parkin or PINK1 deficiency
Source: Mol Neurodegener. 2020 Mar 5;15:17. doi: 10.1186/s13024-020-00363-x (PMC7057660; doi:10.1186/s13024-020-00363-x)
Supplement: Supplementary file 8 — Additional file 7: Table S5. Summary of observed versus expected number of TH-Gal4; parkin null transheterozygotes. [file 13024_2020_363_MOESM7_ESM.docx]

**ADDITIONAL FILE 7:**

Table S5. Summary of observed versus expected number of TH-Gal4; parkin null transheterozygotes.

| **Genotype** | **Total** | **Estimated** | **Observed-1** | **Observed-2** |
| --- | --- | --- | --- | --- |
| TH/TH; parkin^-^/parkin^-^ | 80 | 20 | 0 | 0 |
| TH/TH; parkin^-^/TM6B | 80 | 40 | 22 | 24 |
| TH/TH; TM6B/TM6B | 80 | 20 | 0 | 0 |

Estimated and observed number of flies for the indicated genotypes shown. A total of 80 flies were analyzed in two independent crosses indicated as ‘Observed-1’ and ‘Observed-2’.
