# Supplementary material for: PARIS induced defects in mitochondrial biogenesis drive dopamine neuron loss under conditions of parkin or PINK1 deficiency
Source: Mol Neurodegener. 2020 Mar 5;15:17. doi: 10.1186/s13024-020-00363-x (PMC7057660; doi:10.1186/s13024-020-00363-x)
Supplement: Supplementary file 9 — Additional file 8: Figure S1. Ubiquitous expression of PARIS in Drosophila does not affect 5-HT neurons or cause muscle degeneration. [file 13024_2020_363_MOESM8_ESM.docx]

**ADDITIONAL FILE 8:**

**
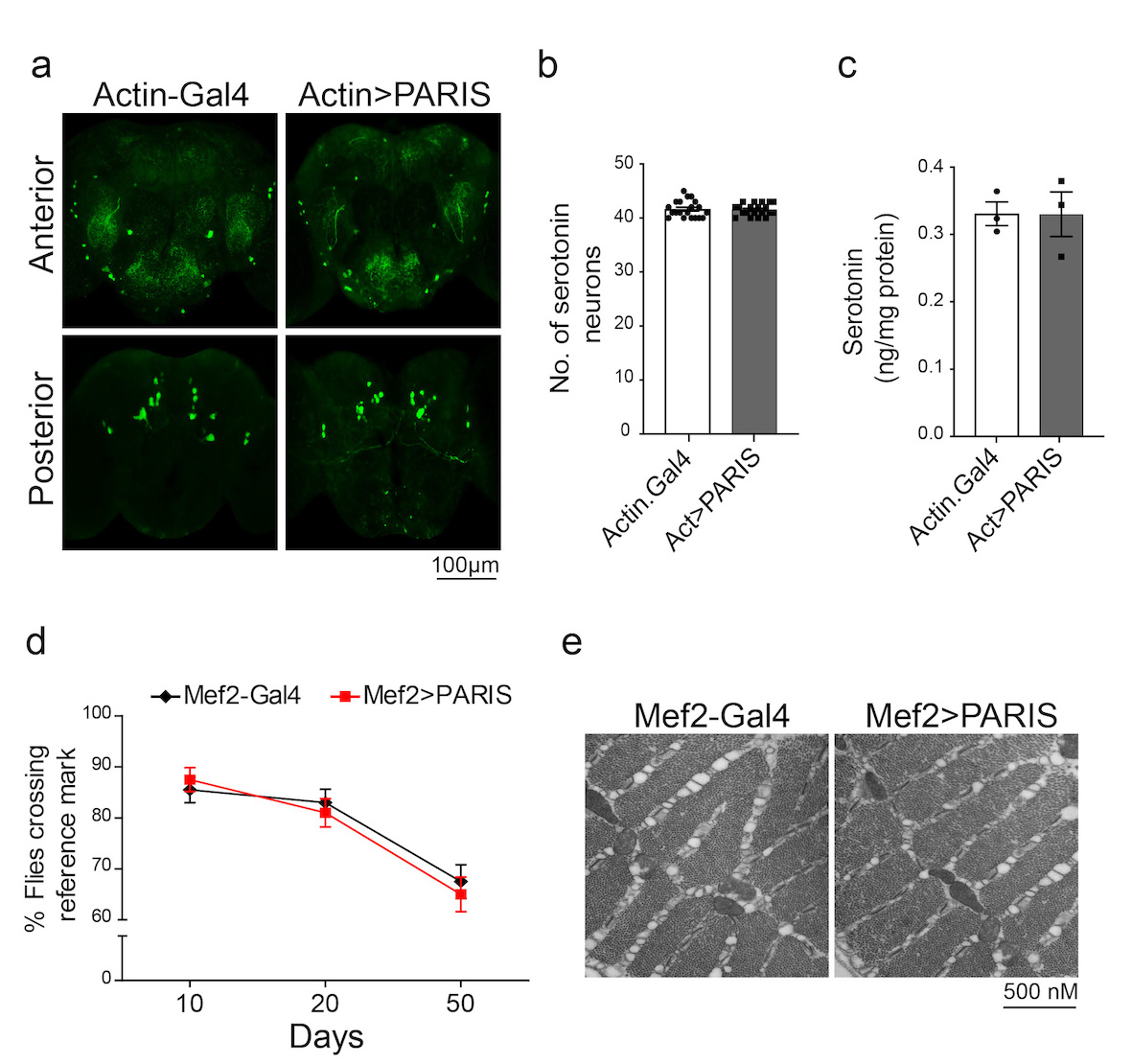
Figure S1. Ubiquitous expression of PARIS in *Drosophila* does not affect 5-HT neurons or cause muscle degeneration.** (a) Representative confocal images of serotonin (5-HT) neurons in *Drosophila* brains of 20-day-old control and PARIS flies stained with an anti-5HT antibody. Scale=100 um. (b) Quantification of number of 5-HT neurons shows no significant difference between control and PARIS flies, N=10. (c) HPLC analysis of 5-HT in fly heads shows no significant difference in 5-HT levels between control and PARIS flies. Quantitative data = mean ± SEM. Unpaired two-tailed Student’s t test, p>0.05. (d) Mef2-Gal4 mediated expression of PARIS in muscles does not cause age-related climbing defects compared to Mef2-Gal4/+ control flies, N=80 flies per genotype. Quantitative data = mean ± SEM. Unpaired two-tailed Student’s t test, p>0.05. (e) Muscle specific expression of PARIS does not cause muscle degeneration as revealed by Scanning Electron Microscopy analysis. Scale = 500 nm. (TIFF)
