# Supplementary material for: PARIS induced defects in mitochondrial biogenesis drive dopamine neuron loss under conditions of parkin or PINK1 deficiency
Source: Mol Neurodegener. 2020 Mar 5;15:17. doi: 10.1186/s13024-020-00363-x (PMC7057660; doi:10.1186/s13024-020-00363-x)
Supplement: Supplementary file 10 — Additional file 9: Figure S2. PARIS induced dopaminergic neurodegeneration rescued by PINK1 and parkin. [file 13024_2020_363_MOESM9_ESM.docx]

**ADDITIONAL FILE 9:
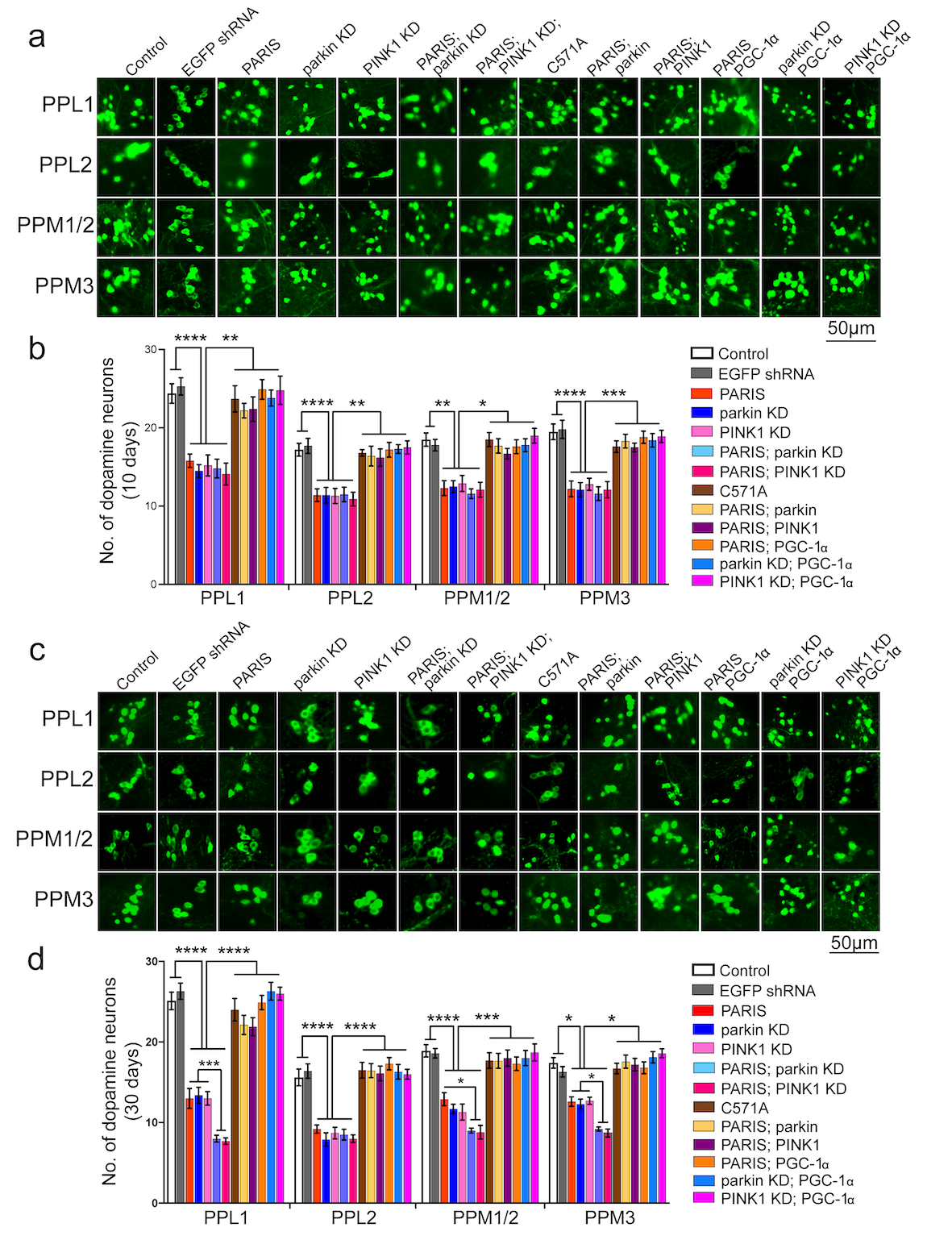
**

**Figure S2. PARIS induced dopaminergic neurodegeneration rescued by PINK1 and parkin.** (A) Representative confocal images of DA neurons in PPL1, PPL2, PPM1/2, and PPM3 DA neuron clusters in the indicated genotypes 10 days post eclosion, Scale=50 μm. (B) Quantification of DA neuron number in PPL1, PPL2, PPM1/2, and PPM3 clusters at 10 days of age. N=10 flies per indicated genotype. (C) Representative confocal images of DA neurons in PPL1, PPL2, PPM1/2, and PPM3 DA neuron clusters in the indicated genotypes 30 days post eclosion. (D) Quantification of DA neuron number in PPL1, PPL2, PPM1/2, and PPM3 clusters at 30 days of age. N=10 flies per indicated genotype. TH-Gal4/+ flies served as control. TH-Gal4 mediated GFP shRNA induction served as non-target control for shRNA response. Quantitative data = mean ± SEM. One-way ANOVA *p<0.05, **p<0.01, ***p < 0.001, ****p < 0.0001. (TIFF)
