# Supplementary material for: PARIS induced defects in mitochondrial biogenesis drive dopamine neuron loss under conditions of parkin or PINK1 deficiency
Source: Mol Neurodegener. 2020 Mar 5;15:17. doi: 10.1186/s13024-020-00363-x (PMC7057660; doi:10.1186/s13024-020-00363-x)
Supplement: Supplementary file 11 — Additional file 10: Table S6. Statistical comparison of total number of DA neurons and climbing performance by genotypes. [file 13024_2020_363_MOESM10_ESM.docx]

**ADDITIONAL FILE 10:**

**Table S6. Statistical comparison of total number of DA neurons and climbing performance by genotypes.**

| **Tukey's multiple comparisons test** | **DA neuron count**  **(Adjusted P-Value)** | | | **Climbing performance  (Adjusted P-Value)** | | |
| --- | --- | --- | --- | --- | --- | --- |
|  | **10 days** | **30 days** | **50 days** | **10 days** | **30 days** | **50 days** |
| Control vs. EGFP shRNA | >0.9999 | >0.9999 | >0.9999 | >0.9999 | 0.9915 | 0.8252 |
| Control vs. PARIS | **<0.0001** | **<0.0001** | **<0.0001** | 0.9536 | **0.0001** | **<0.0001** |
| Control vs. parkin KD | **<0.0001** | **<0.0001** | **<0.0001** | 0.9849 | **0.0021** | **<0.0001** |
| Control vs. PINK1 KD | **<0.0001** | **<0.0001** | **<0.0001** | >0.9999 | **0.0004** | **<0.0001** |
| Control vs. PARIS; parkin KD | **<0.0001** | **<0.0001** | **<0.0001** | **<0.0001** | **<0.0001** | **<0.0001** |
| Control vs. PARIS; PINK1 KD | **<0.0001** | **<0.0001** | **<0.0001** | **0.0002** | **<0.0001** | **<0.0001** |
| Control vs. C571A | 0.9888 | 0.9996 | 0.8209 | >0.9999 | 0.7398 | 0.8545 |
| Control vs. PARIS; parkin | 0.6597 | 0.9956 | 0.9888 | >0.9999 | 0.9755 | 0.2092 |
| Control vs. PARIS; PINK1 | 0.1785 | 0.9289 | 0.9956 | >0.9999 | 0.9327 | >0.9999 |
| Control vs. PARIS; PGC-1α | >0.9999 | >0.9999 | >0.9999 | >0.9999 | 0.9934 | 0.7398 |
| Control vs. parkin KD; PGC-1α | 0.9991 | >0.9999 | >0.9999 | >0.9999 | >0.9999 | 0.9327 |
| Control vs. PINK1 KD; PGC-1α | >0.9999 | 0.9991 | 0.9917 | >0.9999 | 0.9988 | 0.9934 |
| EGFP shRNA vs. PARIS | **<0.0001** | **<0.0001** | **<0.0001** | 0.9836 | 0.0723 | **<0.0001** |
| EGFP shRNA vs. parkin KD | **<0.0001** | **<0.0001** | **<0.0001** | 0.9956 | 0.2863 | **<0.0001** |
| EGFP shRNA vs. PINK1 KD | **<0.0001** | **<0.0001** | **<0.0001** | >0.9999 | 0.1207 | **<0.0001** |
| EGFP shRNA vs. PARIS; parkin KD | **<0.0001** | **<0.0001** | **<0.0001** | **0.0003** | **<0.0001** | **<0.0001** |
| EGFP shRNA vs. PARIS; PINK1 KD | **<0.0001** | **<0.0001** | **<0.0001** | **0.0014** | **<0.0001** | **<0.0001** |
| EGFP shRNA vs. C571A | 0.8996 | 0.9956 | 0.5681 | >0.9999 | >0.9999 | >0.9999 |
| EGFP shRNA vs. PARIS; parkin | 0.3599 | 0.9753 | 0.9151 | >0.9999 | >0.9999 | >0.9999 |
| EGFP shRNA vs. PARIS; PINK1 | 0.0581 | 0.8209 | 0.9517 | >0.9999 | >0.9999 | 0.9607 |
| EGFP shRNA vs. PARIS; PGC-1α | 0.9996 | >0.9999 | >0.9999 | >0.9999 | >0.9999 | >0.9999 |
| EGFP shRNA vs. parkin KD; PGC-1α | 0.9753 | >0.9999 | 0.9956 | >0.9999 | >0.9999 | >0.9999 |
| EGFP shRNA vs. PINK1 KD; PGC-1α | >0.9999 | >0.9999 | 0.9289 | >0.9999 | >0.9999 | >0.9999 |
| PARIS vs. parkin KD | 0.9996 | 0.9996 | >0.9999 | >0.9999 | >0.9999 | >0.9999 |
| PARIS vs. PINK1 KD | >0.9999 | >0.9999 | 0.9753 | 0.9999 | >0.9999 | 0.9988 |
| PARIS vs. PARIS; parkin KD | 0.9517 | **0.0004** | **<0.0001** | **0.0208** | **<0.0001** | **0.0208** |
| PARIS vs. PARIS; PINK1 KD | 0.9151 | **<0.0001** | **<0.0001** | 0.0743 | **<0.0001** | **0.0208** |
| PARIS vs. C571A | **<0.0001** | **<0.0001** | **<0.0001** | 0.7398 | 0.2092 | **<0.0001** |
| PARIS vs. PARIS; parkin | **<0.0001** | **<0.0001** | **<0.0001** | 0.5995 | **0.0404** | **<0.0001** |
| PARIS vs. PARIS; PINK1 | **<0.0001** | **<0.0001** | **<0.0001** | 0.7398 | 0.0743 | **<0.0001** |
| PARIS vs. PARIS; PGC-1α | **<0.0001** | **<0.0001** | **<0.0001** | 0.9755 | **0.0208** | **<0.0001** |
| PARIS vs. parkin KD; PGC-1α | **<0.0001** | **<0.0001** | **<0.0001** | 0.8545 | **0.0021** | **<0.0001** |
| PARIS vs. PINK1 KD; PGC-1α | **<0.0001** | **<0.0001** | **<0.0001** | 0.5995 | **0.0102** | **<0.0001** |
| parkin KD vs. PINK1 KD | >0.9999 | 0.9852 | 0.9852 | >0.9999 | >0.9999 | >0.9999 |
| parkin KD vs. PARIS; parkin KD | >0.9999 | **0.0129** | **<0.0001** | **0.0102** | **<0.0001** | **0.0047** |
| parkin KD vs. PARIS; PINK1 KD | >0.9999 | **0.0019** | **<0.0001** | **0.0404** | **<0.0001** | **0.0047** |
| parkin KD vs. C571A | **<0.0001** | **<0.0001** | **<0.0001** | 0.8545 | 0.5995 | **<0.0001** |
| parkin KD vs. PARIS; parkin | **<0.0001** | **<0.0001** | **<0.0001** | 0.7398 | 0.2092 | **<0.0001** |
| parkin KD vs. PARIS; PINK1 | **<0.0001** | **<0.0001** | **<0.0001** | 0.8545 | 0.3186 | **<0.0001** |
| parkin KD vs. PARIS; PGC-1α | **<0.0001** | **<0.0001** | **<0.0001** | 0.9934 | 0.1286 | **<0.0001** |
| parkin KD vs. parkin KD; PGC-1α | **<0.0001** | **<0.0001** | **<0.0001** | 0.9327 | **0.0208** | **<0.0001** |
| parkin KD vs. PINK1 KD; PGC-1α | **<0.0001** | **<0.0001** | **<0.0001** | 0.7398 | 0.0743 | **<0.0001** |
| PINK1 KD vs. PARIS; parkin KD | 0.9956 | **<0.0001** | **<0.0001** | **0.0009** | **<0.0001** | **0.0004** |
| PINK1 KD vs. PARIS; PINK1 KD | 0.9888 | **<0.0001** | **<0.0001** | **0.0047** | **<0.0001** | **0.0004** |
| PINK1 KD vs. C571A | **<0.0001** | **<0.0001** | **<0.0001** | 0.9934 | 0.3186 | **<0.0001** |
| PINK1 KD vs. PARIS; parkin | **<0.0001** | **<0.0001** | **<0.0001** | 0.9755 | 0.0743 | **<0.0001** |
| PINK1 KD vs. PARIS; PINK1 | **<0.0001** | **<0.0001** | **<0.0001** | 0.9934 | 0.1286 | **<0.0001** |
| PINK1 KD vs. PARIS; PGC-1α | **<0.0001** | **<0.0001** | **<0.0001** | >0.9999 | **0.0404** | **<0.0001** |
| PINK1 KD vs. parkin KD; PGC-1α | **<0.0001** | **<0.0001** | **<0.0001** | 0.9988 | **0.0047** | **<0.0001** |
| PINK1 KD vs. PINK1 KD; PGC-1α | **<0.0001** | **<0.0001** | **<0.0001** | 0.9755 | **0.0208** | **<0.0001** |
| PARIS; parkin KD vs. PARIS; PINK1 KD | >0.9999 | >0.9999 | >0.9999 | >0.9999 | 0.9988 | >0.9999 |
| PARIS; parkin KD vs. C571A | **<0.0001** | **<0.0001** | **<0.0001** | **<0.0001** | **<0.0001** | **<0.0001** |
| PARIS; parkin KD vs. PARIS; parkin | **<0.0001** | **<0.0001** | **<0.0001** | **<0.0001** | **<0.0001** | **<0.0001** |
| PARIS; parkin KD vs. PARIS; PINK1 | **<0.0001** | **<0.0001** | **<0.0001** | **<0.0001** | **<0.0001** | **<0.0001** |
| PARIS; parkin KD vs. PARIS; PGC-1α | **<0.0001** | **<0.0001** | **<0.0001** | **<0.0001** | **<0.0001** | **<0.0001** |
| PARIS; parkin KD vs. parkin KD; PGC-1α | **<0.0001** | **<0.0001** | **<0.0001** | **<0.0001** | **<0.0001** | **<0.0001** |
| PARIS; parkin KD vs. PINK1 KD; PGC-1α | **<0.0001** | **<0.0001** | **<0.0001** | **<0.0001** | **<0.0001** | **<0.0001** |
| PARIS; PINK1 KD vs. C571A | **<0.0001** | **<0.0001** | **<0.0001** | **<0.0001** | **<0.0001** | **<0.0001** |
| PARIS; PINK1 KD vs. PARIS; parkin | **<0.0001** | **<0.0001** | **<0.0001** | **<0.0001** | **<0.0001** | **<0.0001** |
| PARIS; PINK1 KD vs. PARIS; PINK1 | **<0.0001** | **<0.0001** | **<0.0001** | **<0.0001** | **<0.0001** | **<0.0001** |
| PARIS; PINK1 KD vs. PARIS; PGC-1α | **<0.0001** | **<0.0001** | **<0.0001** | **0.0004** | **<0.0001** | **<0.0001** |
| PARIS; PINK1 KD vs. parkin KD; PGC-1α | **<0.0001** | **<0.0001** | **<0.0001** | **<0.0001** | **<0.0001** | **<0.0001** |
| PARIS; PINK1 KD vs. PINK1 KD; PGC-1α | **<0.0001** | **<0.0001** | **<0.0001** | **<0.0001** | **<0.0001** | **<0.0001** |
| C571A vs. PARIS; parkin | 0.9998 | >0.9999 | >0.9999 | >0.9999 | >0.9999 | 0.9988 |
| C571A vs. PARIS; PINK1 | 0.9289 | >0.9999 | >0.9999 | >0.9999 | >0.9999 | 0.9755 |
| C571A vs. PARIS; PGC-1α | 0.9999 | >0.9999 | 0.6597 | >0.9999 | 0.9999 | >0.9999 |
| C571A vs. parkin KD; PGC-1α | >0.9999 | 0.9289 | 0.9969 | >0.9999 | 0.9755 | >0.9999 |
| C571A vs. PINK1 KD; PGC-1α | 0.9517 | 0.8209 | >0.9999 | >0.9999 | 0.9988 | >0.9999 |
| PARIS; parkin vs. PARIS; PINK1 | >0.9999 | >0.9999 | >0.9999 | >0.9999 | >0.9999 | 0.4528 |
| PARIS; parkin vs. PARIS; PGC-1α | 0.9151 | 0.9998 | 0.9517 | 0.9999 | >0.9999 | 0.9999 |
| PARIS; parkin vs. parkin KD; PGC-1α | 0.9956 | 0.8209 | >0.9999 | >0.9999 | 0.9999 | 0.9934 |
| PARIS; parkin vs. pINK1 KD; PGC-1α | 0.4758 | 0.6597 | >0.9999 | >0.9999 | >0.9999 | 0.9327 |
| PARIS; PINK1 vs. PARIS; PGC-1α | 0.4458 | 0.9852 | 0.9753 | >0.9999 | >0.9999 | 0.9327 |
| PARIS; PINK1 vs. parkin KD; PGC-1α | 0.7972 | 0.5064 | >0.9999 | >0.9999 | 0.9988 | 0.9934 |
| PARIS; PINK1 vs. PINK1 KD; PGC-1α | 0.0939 | 0.3331 | >0.9999 | >0.9999 | >0.9999 | 0.9999 |
| PARIS; PGC-1α vs. parkin KD; PGC-1α | >0.9999 | 0.9986 | 0.9986 | >0.9999 | >0.9999 | >0.9999 |
| PARIS; PGC-1α vs. PINK1 KD; PGC-1α | >0.9999 | 0.9888 | 0.9609 | 0.9999 | >0.9999 | 0.9999 |
| parkin KD; PGC-1α vs. PINK1 KD; PGC-1α | 0.9917 | >0.9999 | >0.9999 | >0.9999 | >0.9999 | >0.9999 |

Two-way ANOVA for grouped analysis with Tukey’s post hoc multiple correction shown for comparison of indicated genotypes. Significant values are shown in bold.
