# Supplementary material for: PARIS induced defects in mitochondrial biogenesis drive dopamine neuron loss under conditions of parkin or PINK1 deficiency
Source: Mol Neurodegener. 2020 Mar 5;15:17. doi: 10.1186/s13024-020-00363-x (PMC7057660; doi:10.1186/s13024-020-00363-x)
Supplement: Supplementary file 12 — Additional file 11: Table S7. Statistical comparison of DA neuron number and climbing performance by age groups. [file 13024_2020_363_MOESM11_ESM.docx]

**ADDITIONAL FILE 11:**

**Table S7. Statistical comparison of DA neuron number and climbing performance by age groups.**

| **Genotype** | **Tukey's multiple comparison test** | | | | | |
| --- | --- | --- | --- | --- | --- | --- |
|  | **DA neuron counts**  **(Adjusted P-value)** | | | **Climbing performance**  **(Adjusted P-value)** | | |
|  | **10 vs 30 days** | **10 vs 50  days** | **30 vs 50  days** | **10 vs 30  days** | **10 vs 50  days** | **30 vs 50  days** |
| Control | 0.5177 | **<0.0001** | **<0.0001** | 0.641 | 0.856 | 0.3254 |
| EGFP shRNA | 0.4168 | **<0.0001** | **0.0001** | 0.9416 | 0.0776 | 0.1587 |
| PARIS | **0.0037** | **<0.0001** | **0.0015** | **0.0379** | **<0.0001** | **<0.0001** |
| parkin KD | **0.0037** | **<0.0001** | **0.0319** | 0.1022 | **<0.0001** | **<0.0001** |
| PINK1 KD | **0.0398** | **<0.0001** | **0.0319** | **0.0062** | **<0.0001** | **<0.0001** |
| PARIS; parkin KD | **<0.0001** | **<0.0001** | **<0.0001** | **<0.0001** | **<0.0001** | **0.0062** |
| PARIS; PINK1 KD | **<0.0001** | **<0.0001** | **<0.0001** | **<0.0001** | **<0.0001** | 0.1022 |
| C571A | 0.7542 | **<0.0001** | **<0.0001** | 0.2312 | **0.0118** | 0.4379 |
| PARIS; parkin | 0.9912 | **<0.0001** | **<0.0001** | 0.4379 | **0.0001** | **0.0118** |
| PARIS; PINK1 | 0.9845 | **0.001** | **0.0005** | 0.4379 | 0.3254 | 0.9772 |
| PARIS; PGC-1α | 0.6238 | **<0.0001** | **0.0007** | 0.9772 | **0.0379** | 0.0636 |
| parkin KD; PGC-1α | 0.8257 | **<0.0001** | **<0.0001** | >0.9999 | **0.0379** | **0.0379** |
| PINK1 KD; PGC-1α | 0.9239 | **<0.0001** | **<0.0001** | 0.6923 | **0.0379** | 0.2312 |

Two-way ANOVA for grouped analysis with Tukey’s post hoc multiple correction shown for comparison of indicated age groups. Significant values are shown in bold.
