# Supplementary material for: PARIS induced defects in mitochondrial biogenesis drive dopamine neuron loss under conditions of parkin or PINK1 deficiency
Source: Mol Neurodegener. 2020 Mar 5;15:17. doi: 10.1186/s13024-020-00363-x (PMC7057660; doi:10.1186/s13024-020-00363-x)
Supplement: Supplementary file 13 — Additional file 13 Figure S3. Specific expression of PARIS in Drosophila 5-HT or cholinergic neurons does not cause neuron loss and climbing defects. [file 13024_2020_363_MOESM13_ESM.docx]

**ADDITIONAL FILE 13:**

**
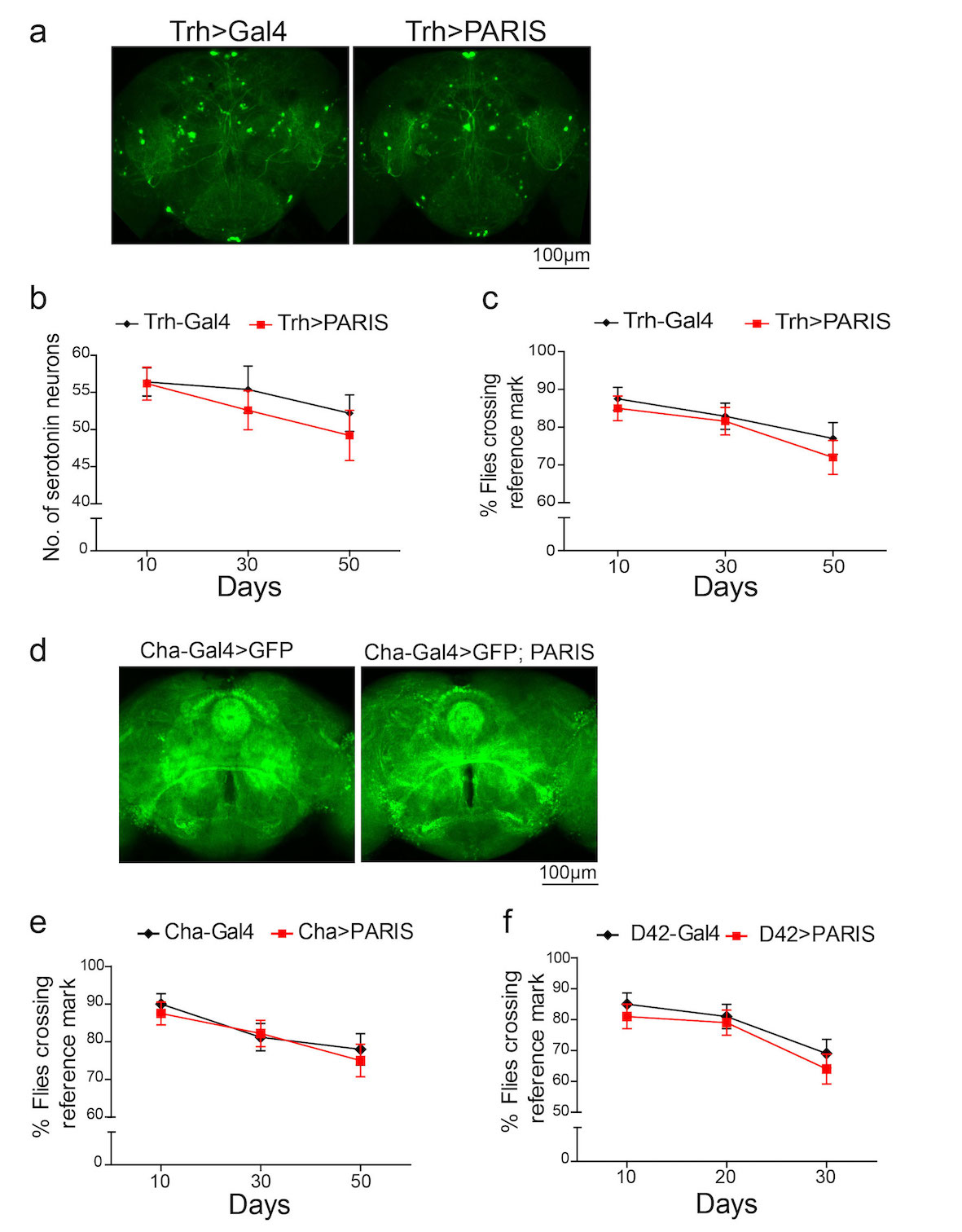
**

**Figure S3. Specific expression of PARIS in *Drosophila* 5-HT or cholinergic neurons does not cause neuron loss and climbing defects.** (a) Representative confocal images showing location of 5-HT neurons in adult *Drosophila* brain visualized by GFP immunofluorescence in 20-day old flies of the indicated genotype (b) Serotonergic expression of PARIS using Trh-Gal4 does not cause progressive loss of 5-HT neurons compared to control (Trh-Gal4>GFP). N=10 flies per indicated genotype. (c) Trh-Gal4 mediated expression of PARIS in 5-HT neurons does not cause progressive decline in climbing performance. N=80 flies per indicated genotype. (d) Representative confocal images of cholinergic neurons visualized by GFP immunofluorescence show overall neuron number unaffected in flies expressing PARIS under the control of Cha-Gal4 driver compared to control flies (Cha-Gal4>GFP). (e) Cha-Gal4 mediated expression of PARIS in cholinergic neurons does not cause age-related climbing defects. N=80 flies per indicated genotype. (f) D42-Gal4 mediated expression of PARIS in motor neurons has no discernible effect on climbing performance compared to control flies (D42-Gal4/+). N=80 flies per indicated genotype. Quantitative data = mean ± SEM. Unpaired two-tailed Student’s t test, p>0.05. (TIFF)
