# Supplementary material for: PARIS induced defects in mitochondrial biogenesis drive dopamine neuron loss under conditions of parkin or PINK1 deficiency
Source: Mol Neurodegener. 2020 Mar 5;15:17. doi: 10.1186/s13024-020-00363-x (PMC7057660; doi:10.1186/s13024-020-00363-x)
Supplement: Supplementary file 14 — Additional file 14: Figure S4. Dopaminergic overexpression of parkin or PINK1 does not abrogate neurotoxicity associated with PARIS phosphomutant. [file 13024_2020_363_MOESM14_ESM.docx]

**ADDITIONAL FILE 14:**

**
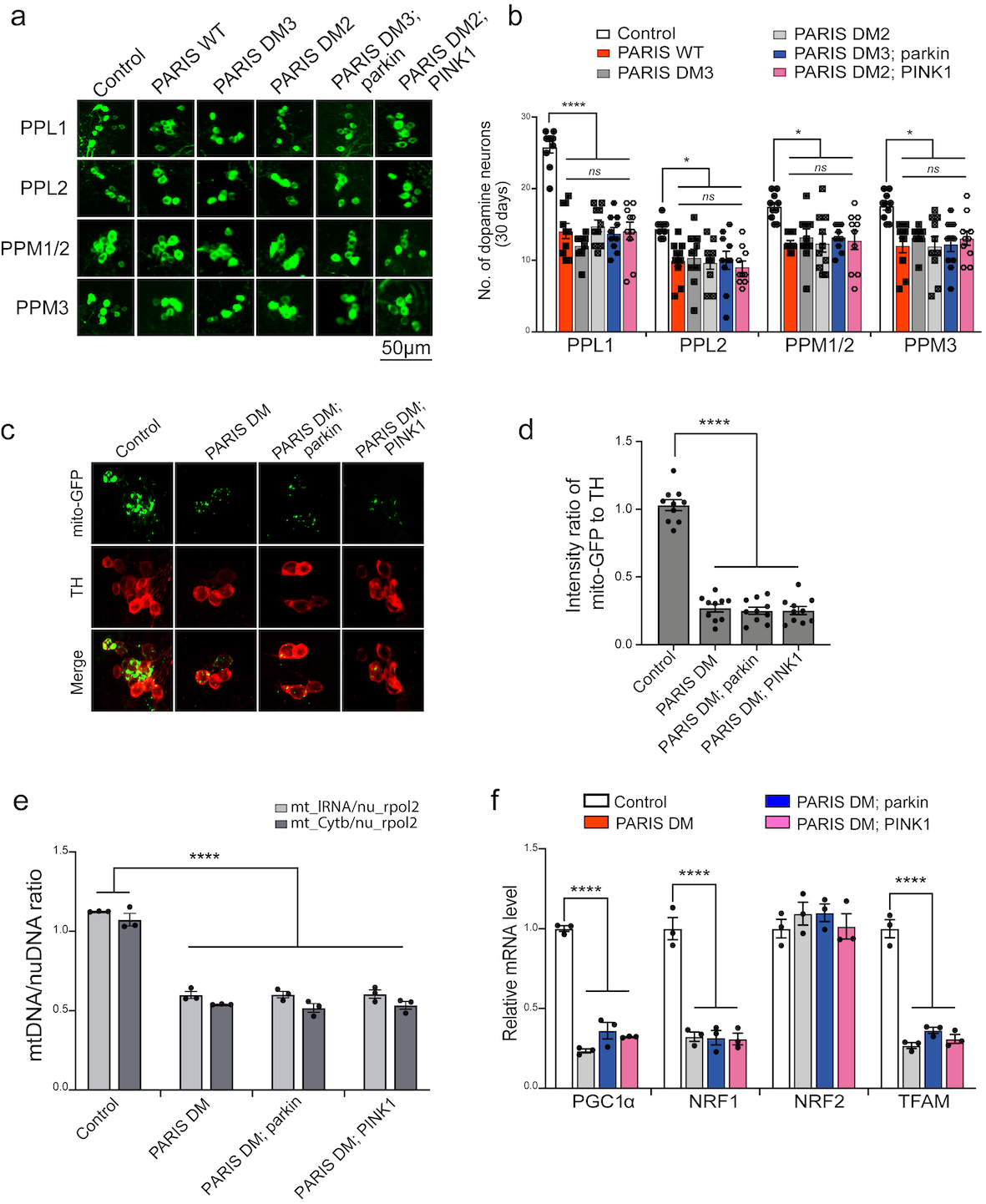
**

**Figure S4. Dopaminergic overexpression of parkin or PINK1 does not abrogate neurotoxicity associated with PARIS phosphomutant.** (a) Representative confocal images of DA neurons in PPL1, PPL2, PPM1/2, and PPM3 DA neuron clusters in the indicated genotypes 30 days post eclosion, Scale 50 μm. (b) Quantification of DA neuron number in PPL1, PPL2, PPM1/2, and PPM3 clusters at 30 days of age. N=10 flies per indicated genotype. TH-Gal4/+ flies served as control. (c) Representative confocal images showing mito-GFP labeling of mitochondria within DA neurons. (d) Quantification of intensity ratio of mito-GFP to TH levels. TH>GFP flies served as control. (e) Quantitative RT-PCR analysis of mitochondrial DNA copy number in FACS sorted DA neurons in 20-day old flies in the genotypes indicated. TH>GFP flies served as control. Mean of three independent FACS experiments each employing 50 fly brains for the indicated genotypes depicted. (f) Quantitative RT-PCR analysis of transcript levels of *Drosophila* homologs of PGC-1α (Spargel), NRF1 (ewg), NRF-2 (Delg) and mitochondrial transcriptional factor A (TFAM) in FACS sorted DA neurons from 20-day old flies. TH>GFP flies served as control. Mean of three independent FACS experiments each using 50 fly brains per genotype shown. Quantitative data = mean ± SEM. One-way ANOVA *p<0.05, ****p < 0.0001. (TIFF)
