# Supplementary material for: PARIS induced defects in mitochondrial biogenesis drive dopamine neuron loss under conditions of parkin or PINK1 deficiency
Source: Mol Neurodegener. 2020 Mar 5;15:17. doi: 10.1186/s13024-020-00363-x (PMC7057660; doi:10.1186/s13024-020-00363-x)
Supplement: Supplementary file 15 — Additional file 15: Figure S5.Drosophila homolog of PARIS causes age-dependent loss of DA neurons and climbing defects. [file 13024_2020_363_MOESM15_ESM.docx]

**ADDITIONAL FILE 15:**

**
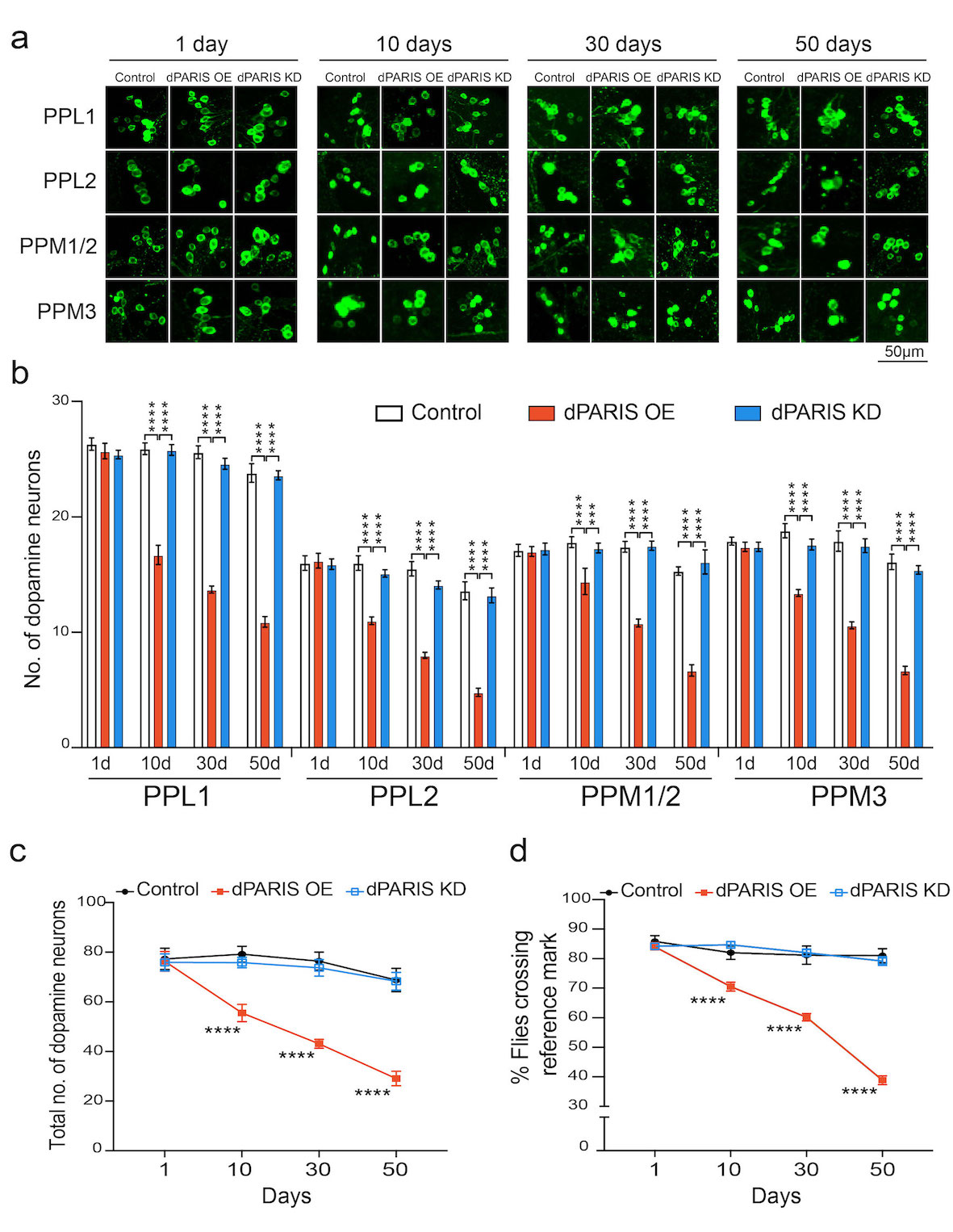
**

**Figure S5. *Drosophila* homolog of PARIS causes age-dependent loss of DA neurons and climbing defects.** (a) Representative confocal images of individual DA neurons in PPL1, PPL2, PPM1/2, and PPM3 DA neuron clusters in the indicated genotypes on 1, 10, 30- and 50-days post eclosion. Scale 50 μm. (b) Quantification of DA neuron numbers in PPL1, PPL2, PPM1/2, and PPM3 clusters for the indicated genotypes at the different time points, N=10 flies per genotype. Quantitative data = mean ± SEM. One-way ANOVA ***p < 0.001, ****p < 0.0001. (c) Quantification of total number of DA neurons at the indicated time points show progressive loss of DA neurons in flies expressing dPARIS under the control of TH-Gal4 driver. TH-Gal4 driven knockdown (KD) of dPARIS exhibits neuron loss comparable to control (TH-Gal4/+). N=10 flies per indicated genotype. Quantitative data = mean ± SD. One-way ANOVA ****p < 0.0001. (d) Climbing performance in TH>dPARIS flies shows age-dependent decline compared to TH>dPARIS KD and control flies (TH-Gal4/+). N=60 flies per indicated genotype. Quantitative data = mean ± SEM. One-way ANOVA ****p < 0.0001. (TIFF)
