# Supplementary material for: PARIS induced defects in mitochondrial biogenesis drive dopamine neuron loss under conditions of parkin or PINK1 deficiency
Source: Mol Neurodegener. 2020 Mar 5;15:17. doi: 10.1186/s13024-020-00363-x (PMC7057660; doi:10.1186/s13024-020-00363-x)
Supplement: Supplementary file 16 — Additional file 16: Figure S6. Dopaminergic neurotoxicity resulting from reduced parkin or PINK1 activity prevented under conditions of dPARIS knockdown. [file 13024_2020_363_MOESM16_ESM.docx]

**ADDITIONAL FILE 16:**

**
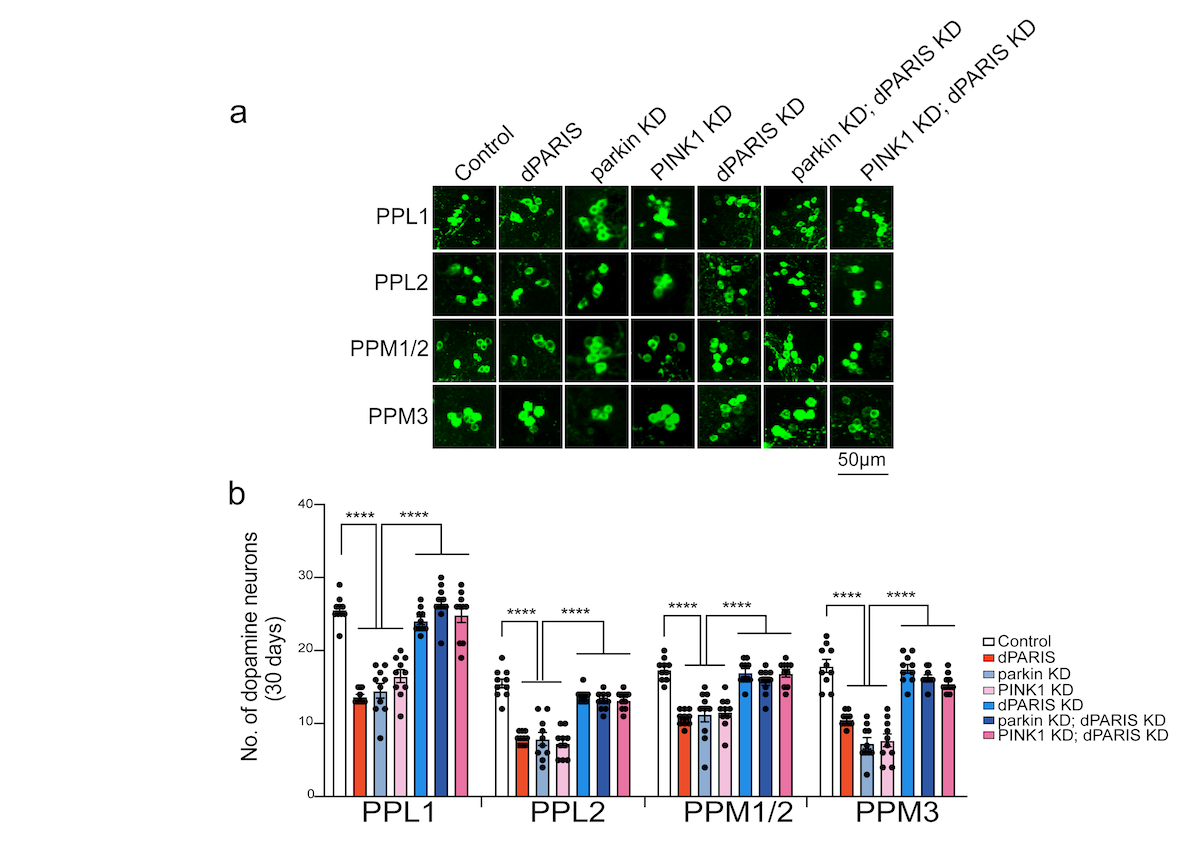
**

**Figure S6. Dopaminergic neurotoxicity resulting from reduced parkin or PINK1 activity prevented under conditions of dPARIS knockdown.** (a) Representative confocal images of DA neurons in PPL1, PPL2, PPM1/2, and PPM3 DA neuron clusters in the indicated genotypes 30 days post eclosion, Scale 50 μm. (b) Quantification of DA neuron number in PPL1, PPL2, PPM1/2, and PPM3 clusters at 30 days of age. N=10 flies per indicated genotype. TH-Gal4/+ flies served as control. Quantitative data = mean ± SEM. One-way ANOVA ****p < 0.0001. (TIFF)
